# Collagen microarchitecture from polarized light imaging: a biomechanics perspective

**DOI:** 10.1117/1.JBO.31.1.010902

**Published:** 2026-01-13

**Authors:** Miriam Bohlmann Kunz, Po-Yi Lee, Gaël Latour, Bin Yang, Marie-Claire Schanne-Klein, Kazuhiro Kurokawa, Ian A. Sigal

**Affiliations:** a University of Pittsburgh, School of Medicine, Laboratory of Ocular Biomechanics, Department of Ophthalmology, Pittsburgh, Pennsylvania, United States; b Massachusetts General Hospital, Wellman Center for Photomedicine, Boston, Massachusetts, United States; c Institut Polytechnique de Paris, Ecole Polytechnique, CNRS, Inserm, Laboratoire d’Optique et Biosciences, Palaiseau, France; d Université Paris-Saclay, Gif-sur-Yvette, France; e Duquesne University, School of Science and Engineering, Department of Biomedical Engineering, Pittsburgh, Pennsylvania, United States; f Devers Eye Institute, Legacy Research Institute, Legacy Health, Discoveries in Sight Research Laboratories, Portland, Oregon, United States; g University of Pittsburgh, Swanson School of Engineering, Department of Bioengineering, Pittsburgh, Pennsylvania, United States

**Keywords:** polarized light microscopy, biomechanics, collagen, crimp, deformation

## Abstract

**Significance:**

Collagen, the main load-bearing component in tissue, is present in all animals and forms a variety of networks from the fibrils, fibers, bundles, and lamellae into which it self-assembles. The collagen microstructure is different among tissue types, and the different microstructures give rise to tissue-specific mechanical properties. Therefore, methods for visualizing collagen fibers and their orientation are essential for understanding the biomechanical properties of tissue.

**Aim:**

Our aim in this review is to provide the basis for understanding the methodology of polarized light imaging methods and how they can be used to characterize collagen microstructure.

**Approach:**

We begin with a description of collagen microstructure and its relationship to tissue biomechanics, a basic formalism of polarized light, and how collagen interacts with polarized light. We then describe polarized light microscopy and its various forms, particularly instant polarized light microscopy, then polarization-sensitive optical coherence tomography, and last, polarization-resolved second-harmonic generation microscopy.

**Results:**

We describe methods for imaging collagen microstructure with polarized light from *in vivo* methods to high-resolution volumetric imaging of tissue sections.

**Conclusions:**

We intend to help those interested in using polarized light to image and understand the relationship between collagen microstructure and biomechanics.

## 1 Introduction

Collagen is the most abundant protein in mammals and is the main load-bearing component in soft tissue. Collagen can form a vast variety of networks from fibrils that are 10 to 100s of nanometers in diameter and can spontaneously self-assemble to form fibers and other structures.^1^^–^^3^ Characterizing the collagen microarchitecture in tissue requires a method with high spatial resolution that can distinguish collagen from other tissue components. Polarized light imaging can do this by capitalizing on the intrinsic optical properties of collagen, particularly its birefringence and being noncentrosymmetric. Hence, several imaging techniques have been adapted to use polarized light, from relatively basic brightfield imaging and polarized light microscopy (PLM),^4^^–^^9^ to more complex second-harmonic generation (SHG) imaging,^10^^–^^14^ and optical coherence tomography (OCT).^15^^–^^22^ The combined techniques have improved selectivity and resolution, revealing details of the shape and architecture of the collagen, such as fiber crimp and interweaving.^23^^,^^24^ In the more recent implementations, such as instant polarized light microscopy (IPOL), the imaging can be done at frame-rate speed. This is not just convenient, but it opens the door to visualizing dynamic processes, such as the microstructural changes associated with tissue distortion.^25^ Polarization-resolved second-harmonic generation microscopy (pSHG) and polarization-sensitive OCT (PS-OCT) can use optical sectioning to enable 3D reconstruction of collagen microarchitecture. Altogether, these techniques provide crucial information on tissue structure and mechanics previously out of reach.

Our goal with this review is to provide an introduction to the powerful techniques available for studying collagen microstructure and biomechanics with polarized light, including examples from more conventional tissue section microscopy, high-resolution volumetric imaging, and *in vivo* imaging. We start with a description of various aspects of collagen microstructure architecture, categorizing the features into four aspects: density, anisotropy, interweaving, and crimp, and we will discuss their relevance to biomechanical properties. Next, we give a description of polarized light and material properties that can affect the polarization of light. We then describe multiple polarized light imaging methods, starting with PLM, including several of its variations (e.g., cPLM, IPOL, and SPLM), followed by PS-OCT, and finally a brief description of pSHG.

It is important to acknowledge that the use of polarized light to characterize tissues has a long history. Many techniques have been reported by multiple laboratories, which has led to a confusing naming landscape. Reports have used different names to describe what seems like the same technique, or conversely, used the same name for techniques that are not identical. For our research labs, feedback steered us to name the techniques we use based on the applications in the context of soft tissue biomechanics. Although this may have helped some readers, we acknowledge that other readers may know the techniques by other names. We encourage readers to carefully look at the references and remain alert that technique names are not final or definitive. Furthermore, there are many variations of techniques using polarized light that are adapted and applied for many purposes. We aimed to provide a broad perspective, including the most popular techniques applied to soft tissue biomechanical characterization, and therefore, there will be techniques that we do not cover. Nevertheless, we anticipate that by covering the fundamentals of soft tissue microstructure and polarized light, readers will be well equipped to understand other techniques.

## 2 Motivating Tissue Microstructure Quantification

Tissue microstructure determines the mechanical properties, such as hardness and elastic modulus, of the tissue. A major extracellular component and determinant of microstructure in tissue is collagen, which is especially present in tendons, ligaments, bones, skin, and eyes.^26^ The arrangement of collagen and the general microstructure vary among tissue types, can be heterogeneous within individual tissues, and can be affected by aging and disease. To fully understand aging and disease mechanisms, it is crucial to characterize and determine the role of tissue microstructure with the associated pathological changes. One function of collagen is that it bears tensile force during stretch, resulting in a vast interest in the role of collagen microstructure on the stretching of tissue. The general impact of collagen microstructure on the mechanical behavior of soft tissues has been investigated for decades.^26^^–^^38^

**Fig. 1:**
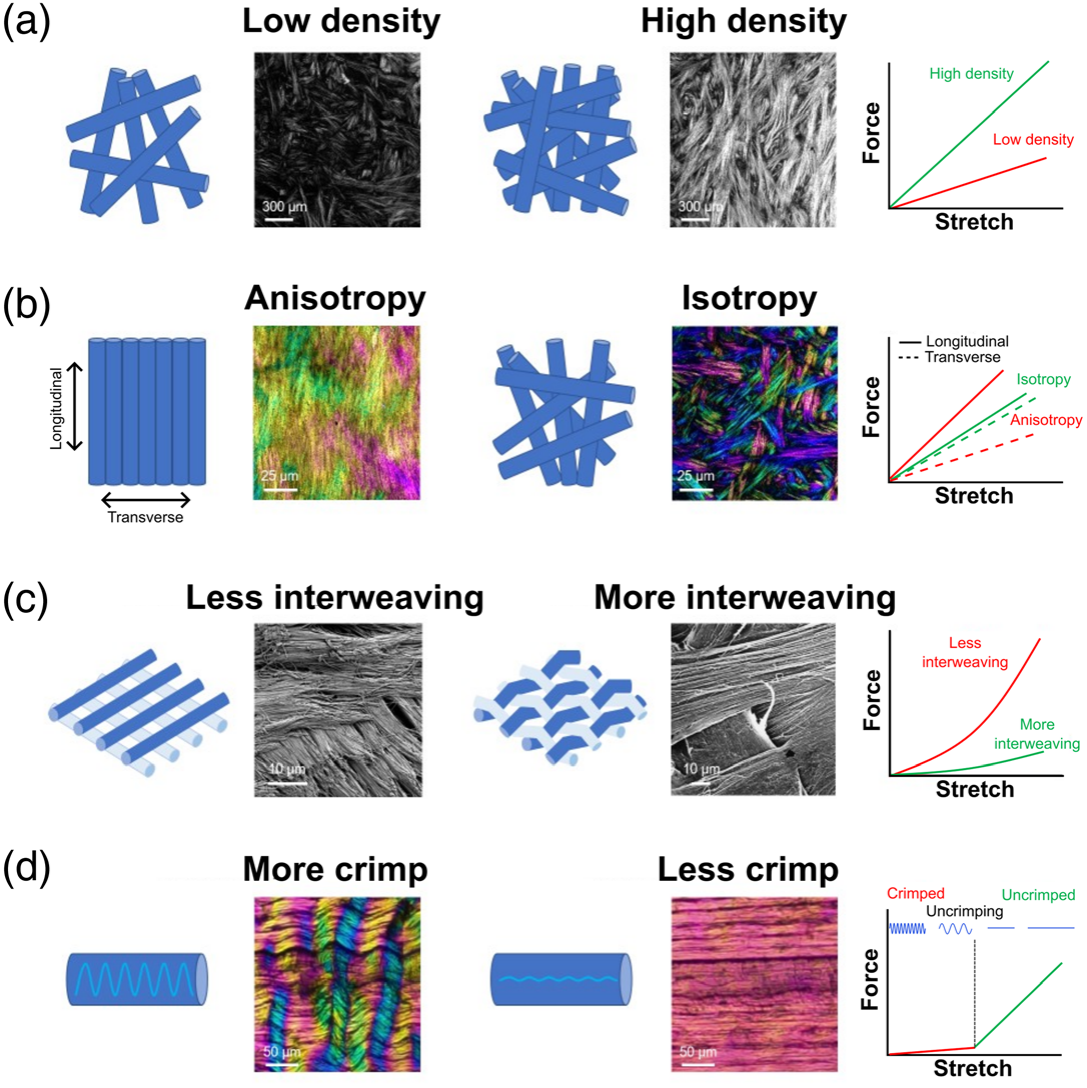
Four key properties of collagen microstructure and their effects on biomechanics. (a) Fiber density: IPOL images of sclera, in which brightness is proportional to local fiber density.^41^ Modulus of elasticity increases with collagen density.^42^ (b) Anisotropy: IPOL images of scleral sections where color represents local fiber orientation.^41^ The force-stretch curve of an isotropic structure falls between those of the anisotropic structure.^43^ (c) Interweaving: SEM images show that collagen fibrils exhibit less interweaving in a cow sclera than in the human corneal stroma. The full effects of soft tissue fiber interweaving are complex and difficult to predict, but they are generally expected to reduce stiffness and increase strength.^24^ (d) Crimp: IPOL images of chicken tendon sections, where color bands indicate that the collagen fibers are crimped. As a beam stretches, the collagen fibers uncrimp, requiring relatively little force until the fiber has lost all crimp and is straight. The straightened collagen fibers can be stretched further only by making the fibers longer, which requires a larger and increasing force; thus, the beam appears stiffer. Panel in row C, column 4 adapted from Ref. 23 with permission from Pergamon. Panels in row D, columns 2 and 4 adapted from Ref. 41.

The major load-bearing units of tissue are the fibrils, fibers, and fiber bundles into which collagen self-assembles. The arrangement or architecture of these fibers and bundles dictates the mechanical properties of soft tissues.^1^^,^^39^^,^^40^ Over the course of the decades of studies into the relationship between collagen microstructure and soft tissue mechanical properties, four aspects of collagen fiber arrangement have emerged as determinants of mechanical behavior under stretch: density, anisotropy, interweaving, and fiber crimp. These four aspects are illustrated in Fig. 1, and we will briefly discuss the contribution of these four properties to collagen mechanics.

The first property we consider is density. Intuitively, the more collagen there is per unit volume, the stiffer the tissue.^42^ Simply put, it requires more force to deform the tissue when there is more structural material present, i.e., collagen. This principle is also true of fiber bundles; bundles with a larger diameter are stiffer and provide more support to adjacent tissue. The next property we consider is anisotropy, or the extent to which the fibers are aligned in the same direction. In the extreme isotropic case, where the fibers are oriented randomly, the linear modulus and failure strength increase linearly with increased density.^42^ When the fiber orientation is anisotropic, known as structural anisotropy, the mechanical properties are also anisotropic. In general, anisotropic tissues are stiffer in the direction that the fibers are aligned (longitudinal) than in the transverse direction [Fig. 1(b)].^43^ Anisotropy should be considered in three dimensions as fiber networks are not just two-dimensional. Tissue may be isotropic in a plane but have different organization in 3D. The third property we consider is interweaving. Interweaving occurs, for example, in the cornea and the skin. In the cornea, 300 to 500 lamellae cross and interweave at various angles, with increasing interweaving in the anterior and peripheral cornea than in the central and posterior cornea.^23^^,^^44^ Interwoven fibers must have undulations that reduce their load-bearing ability compared with straight fibers, although this can be somewhat compensated by interlocking. Interweaving also transfers loads between fibers, which increases tissue strength.^24^^,^^45^ Overall, interweaving has strong effects on soft tissue mechanics that are not yet fully understood, mainly because of the paucity of suitable experimental techniques to study them.

The last property we consider is crimp. As already mentioned, collagen is the main load-bearing component of soft tissue. When collagen fibers are unloaded, they can appear buckled or crimped [Fig. 1(d)]. This crimp usually occurs at the level of aggregated fibers such as in the tendon,^46^ rabbit corneal stroma,^47^ lamina cribrosa beams,^48^ or sclera fibers.^49^ The force required to progressively straighten and stretch a crimped collagen fiber, also called recruitment, is nonlinear and is also a primary reason why soft tissues have a nonlinear elastic response. At first, very little force is required to elongate the fiber until all the crimp is gone. Then, once the fiber is uncrimped, more force is required to stretch the fiber.^50^ The process of recruitment can vary within and between tissues, which also leads to variation in mechanical behavior. That is, after a certain amount of stretch is applied, certain fibers will be under greater loads than others, partially depending on the amount of crimp in the fibers. One example of this is in the lamina cribrosa, which is made of collagen fiber beams of various widths. It was predicted that the narrower beams would be weaker than the wider ones. However, this proved not to be the case. It was visualized with PLM that the wider beams had more crimp than the narrower ones, and thus, more fibers in the narrower beams would be recruited at the same level of stretch.^48^ This example illustrates the ability of PLM to characterize the fiber microarchitecture with high spatial resolution. A combination of PLM with mechanical testing can yield further insight into the relationship between microarchitecture and tissue mechanics.

The properties of collagen architecture are not independent of each other. For example, interweaving is more likely for high-density isotropic collagen networks than for either low-density or anisotropic networks. In addition, we have only discussed four properties of collagen fiber architecture, but there are more properties that can be considered, such as the type of collagen or the number of fibrils forming a single fiber. The complexity of collagen fiber architecture and its relationship to mechanical behavior underline the need for methods that can visualize collagen fibers and their orientation.

## 3 Theory

### 3.1 Formalism of Polarized Light

What makes PLM different from standard brightfield microscopy is the use of polarized light. In this section, we will provide a brief description of polarized light, as well as a description of how matter can affect the polarization of light. Further description of polarized light and its properties can be found in Refs. 51 and 52.

Electromagnetic radiation, or light, is composed of an oscillating electric and magnetic field that are perpendicular to each other and the propagation of the light. The polarization of light refers to the electric field vector as a function of time at a fixed point. The magnitude and direction of the electric field of unpolarized light are random. Light can be linearly polarized, which means that the direction and magnitude of the electric field are constant; circularly polarized, which means the direction is periodic and the magnitude is constant; or elliptically polarized, which means the direction and magnitude are periodic. Linear and circularly polarized light can be seen as special cases of elliptically polarized light. Linear polarizations can be seen as the eigenvectors of polarized light, and all polarizations can be expressed as a linear combination of linear polarizations at the same frequency of oscillation.

**Fig. 2:**
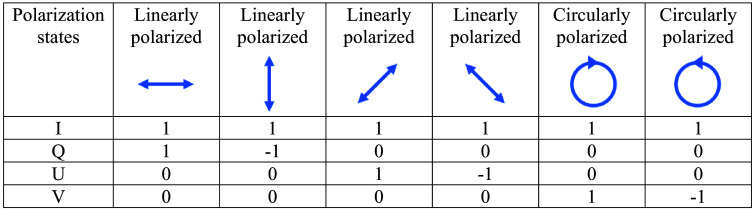
Stokes vectors for degenerate polarization states.

Two major methods for representing the polarization state of light exist, Jones and Stokes vectors. In this section, we describe Stokes vectors as they are a rigorous method to represent polarized light. In our description of PS-OCT, we briefly describe Jones vectors. The Stokes vector, S, has four values:

$$S=(\begin{array}{c} I\\ Q\\ U\\ V \end{array}),$$ (1)

where I is the intensity, Q is the shape of the polarization ellipse, U is the orientation of the polarization ellipse, and V is the chirality of the polarization ellipse. The Stokes vectors for special cases are listed in Fig. 2.

Just as the polarization of light can be expressed with a Stokes vector, the effect of an optical component on the polarization can be expressed mathematically with a transformation matrix known as a Mueller matrix. The Mueller matrix is a 4×4 matrix in which all the elements are real and independent. The Mueller matrix of a component operates on the Stokes vector of the incoming light, resulting in the Stokes vector of the outgoing light:

$$S_{\mathrm{out}}={MS}_{\mathrm{in}}.$$ (2)

If the light passes through multiple polarizing elements, $M_{1}$, $M_{2}$, and $M_{3}$, the total effect of all three can be written as

$$M_{\mathrm{eff}}=M_{1}\cdot M_{2}\cdot M_{3}.$$ (3)

The operation of the matrices is not commutative. Mueller calculus allows the simulation of the polarization of light after interacting with various and multiple polarizing optics.

### 3.2 Effects of Collagen on the Polarization of Light

A Mueller matrix can also be used to approximate the effect of the sample on the polarization of light in a polarization measurement.^53^ Therefore, it is possible to simulate the result of a polarized light experiment for a sample with known optical properties. For instance, biological tissue exhibits a feature called birefringence.^53^^–^^57^ In this section, we will describe the birefringent optical property of collagen and its effect on polarized light.

The refractive index is a complex value property of a material that governs its interaction with light. The real part of the refractive index is the ratio of the speed of light traveling through the given material to the speed of light in a vacuum and can be wavelength dependent. Just like materials can have structural anisotropy, they can also have optical anisotropy, where the refractive index is different depending on the orientation of the material relative to the polarization and propagation of the light. The property of a material having optical anisotropy with different refractive indices is called birefringence. Numerically, the birefringence of uniaxial material is defined as the difference between the two refractive indices, called the ordinary ($n_{o}$) and extraordinary ($n_{e}$) indices

$$\Delta n=n_{e}-n_{o}.$$ (4)

Experimentally, the birefringence of many collagenous tissues such as tendon, sclera, cartilage, and skin is on the order of $10^{-3}$.^58^^–^^60^ Both the molecular structure and microstructure of a material can contribute to its birefringence, called intrinsic and form birefringence.^7^ Intrinsic birefringence occurs from the spatial arrangement of the atoms or molecules in the material such that there is an anisotropic distribution of electric charge.^61^ Form birefringence results from the ordered spatial arrangement of micro-objects into a medium with a different refractive index.^62^^,^^63^ Summing the intrinsic and form birefringence gives the total birefringence

$$\Delta n_{\mathrm{comp}}=\Delta n_{\mathrm{intr}}+\Delta n_{\text{form}}.$$ (5)

**Fig. 3:**
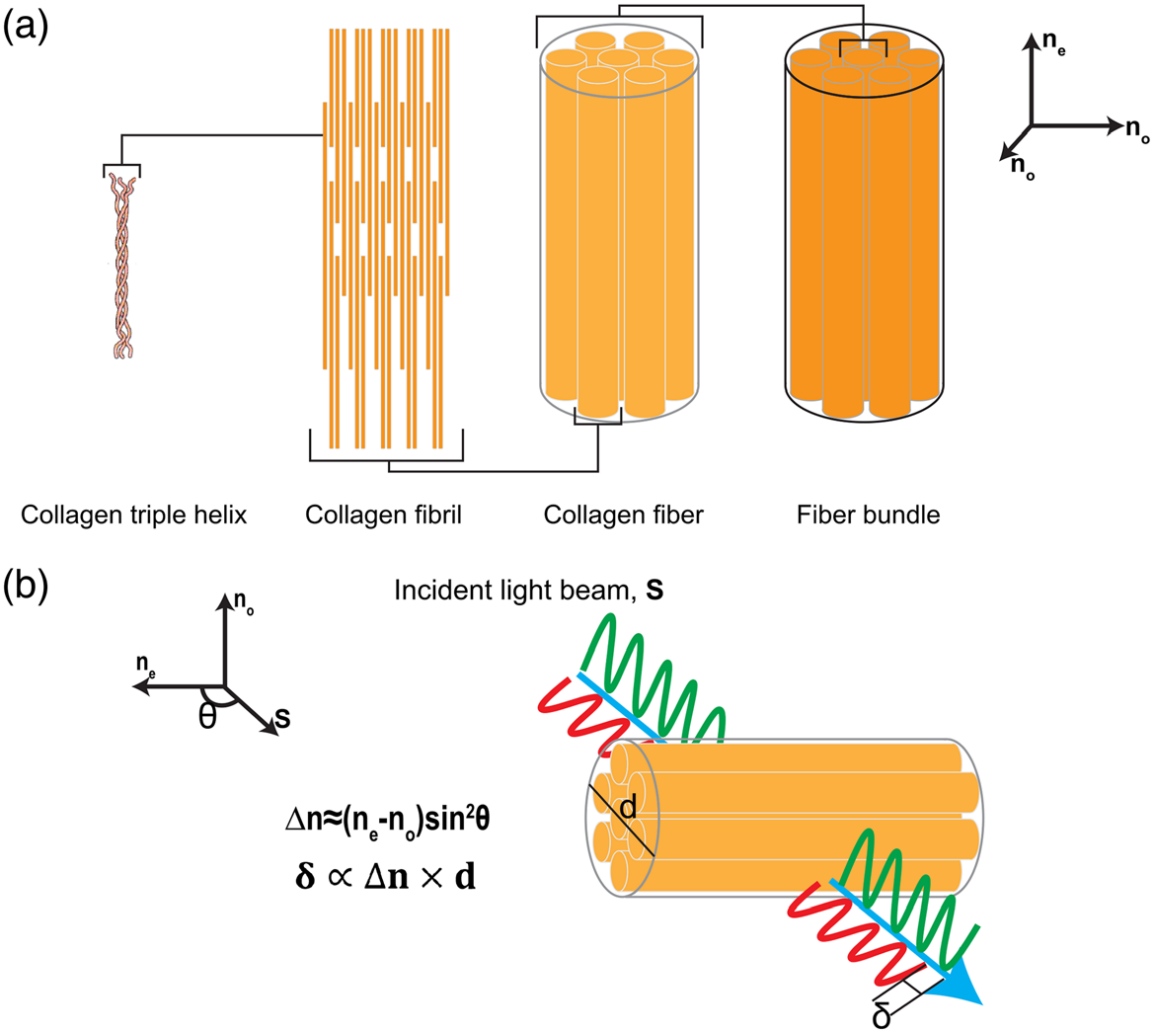
Collagen hierarchical structure and optic axes orientations. (a) Triple helix structure of a single collagen molecule, which forms fibrils, which self-assemble into fibers and fiber bundles. The ordinary refractive index is perpendicular to the long fiber axis, and the extraordinary refractive index is parallel to the long axis of the fiber. (b) The birefringence, Δn, is dependent on the difference between the extraordinary and ordinary axis and the angle between the propagation of the light and the optic axis of the collagen fibers. After passing through the material, the phase retardance between the light along the extraordinary and ordinary axes is proportional to the birefringence and the thickness of the material.

Collagen fibers have both intrinsic and form birefringence. The smallest structural unit of collagen is a triple helix made of three polypeptide chains. This single collagen molecule is noncentrosymmetric and is intrinsically birefringent. These molecules self-assemble into fibrils, which then form cylindrical collagen fibers, with the long axis of the triple helix aligned. This cylindrical structure immersed in an isotropic ground substance leads to the form birefringence of collagen (Fig. 3), with the extraordinary refractive index parallel to the cylindrical axis of the fibers and the ordinary refractive index perpendicular to the cylindrical axis.^64^^,^^65^ Light that is propagating parallel to the optic axis is only governed by the ordinary refractive index. Light with any other propagation direction is governed by both the ordinary and extraordinary refractive indices. The form birefringence can be described as^55^

$$\Delta n_{\text{form}}=n_{e}-n_{o}=\frac{f_{1}f_{2}(n_{1}-n_{2}{)}^{2}}{f_{1}n_{1}+f_{2}n_{2}},$$ (6)

where $f_{1}$ and $f_{2}$ are the volume fractions of the cylinders and the ground substance, respectively, and $n_{1}$ and $n_{2}$ are the corresponding refractive indices. Equation (6) assumes that the wavelength of light is much larger than the diameter of the cylinder, called the Rayleigh limit. At the other extreme, when the diameter of the cylinder is large compared with the wavelength of light, the birefringence approaches zero.

The orthogonal polarization components parallel to the ordinary or extraordinary axes pass through uniaxial birefringent materials at different speeds, resulting in phase retardance (or phase shift) between the two components. The phase retardance, δ, is proportional to the birefringence and the distance of material the light passes through, d. The phase retardance for a given wavelength of light, λ, can be expressed as^66^

$$\delta =\frac{2\pi \Delta nd}{\lambda }=\frac{2\pi (n_{e}-n_{o})}{\lambda }d.$$ (7)

We note that the phase retardance is dependent on the wavelength of light, a point which will be utilized in IPOL.

The extent of phase retardance is dependent on the orientation of the birefringent material relative to the direction of propagation of the light. If the direction of propagation of the light is not perpendicular to the optic axis, the effective birefringence decreases, such that^67^

$$\Delta n_{\mathrm{eff}}=\frac{n_{o}n_{e}}{\sqrt{n_{o}^{2}\;\sin^{2}\;\theta +n_{e}^{2}\;\cos^{2}\;\theta }}-n_{o},$$ (8)

and for a small refractive index, the effective refractive index can be approximated as^68^

$$\Delta n_{\mathrm{eff}}\approx (n_{e}-n_{o})\sin^{2}\;\theta =(n_{e}-n_{o})\cos^{2}\;\psi ,$$ (9)

where θ is the angle between the optic axis and the direction of the light propagation, and ψ is the out-of-plane angle. The out-of-plane angle is the angle between the optic axis and the imaging plane. For a given effective refractive index, the effective retardance can be written as^69^

$$\delta_{\mathrm{eff}}(\psi )=\frac{2\pi \Delta n_{\mathrm{eff}}d}{\lambda }=\frac{2\pi (n_{e}-n_{o})d}{\lambda }\;\cos^{2}\;\psi .$$ (10)

The premise of polarization microscopy for imaging biological tissue is to capitalize on the birefringence of collagen and the orientational dependence of the phase retardance to determine the orientation of collagen fibers.

## 4 Methods of Polarization Microscopy

Polarization microscopy is a method that has been used in biological imaging for almost 100 years.^70^^,^^71^ Polarization microscopy can visualize optically anisotropic materials, such as collagen, that might otherwise not be observable via traditional brightfield imaging because the material is optically transparent or has a similar absorptive profile to the surrounding material. Developments in the past 100 years, and particularly within the past decade, have enabled qualitative and quantitative polarization methods to visualize the orientation of collagen fibers in tissue. In this section, we describe the experimental methods for polarization microscopy and the benefits associated with each. First, we describe methods derived from brightfield imaging: cross-polarized light microscopy (cPLM), quantitative PLM, IPOL, a variation of IPOL called IPOLπ, and structured polarized light microscopy (SPLM). Then, we describe polarization-sensitive optical coherence tomography (PS-OCT), which has applications for *in vivo* imaging of collagen architecture. Last, we provide a brief description of pSHG. Some basic features and limitations of these methods are listed in Table 1.

**Table 1:** Polarization microscopy techniques for collagen tissue assessment.

	Features	Assessment methods	Limitations
cPLM	Simplest setup	Specimen rotation	Limited quantitative information
PLM	Accurate quantification	Arithmetic calculation of polarization images	Slow imaging speed; sensitive to noise
IPOL	Real-time and single-image quantification	Interpolation via color in images	Indistinguishable color for orthogonal fibers
IPOLπ	Same as IPOL and can distinguish orthogonal fibers	Interpolation via color in images	Limited to thin tissue sections
SPLM	Thick tissue imaging	Arithmetic calculation of fringe-pattern polarization images	Slow imaging speed
PS-OCT	*In vivo* volumetric imaging	Arithmetic reconstruction of depth-resolved polarization images	Speckle noise; slow imaging speed
pSHG	High-resolution volumetric imaging	Arithmetic reconstruction of depth-resolved polarization images	Slow imaging speed; expensive

### 4.1 Cross-Polarized Light Microscopy

**Fig. 4:**
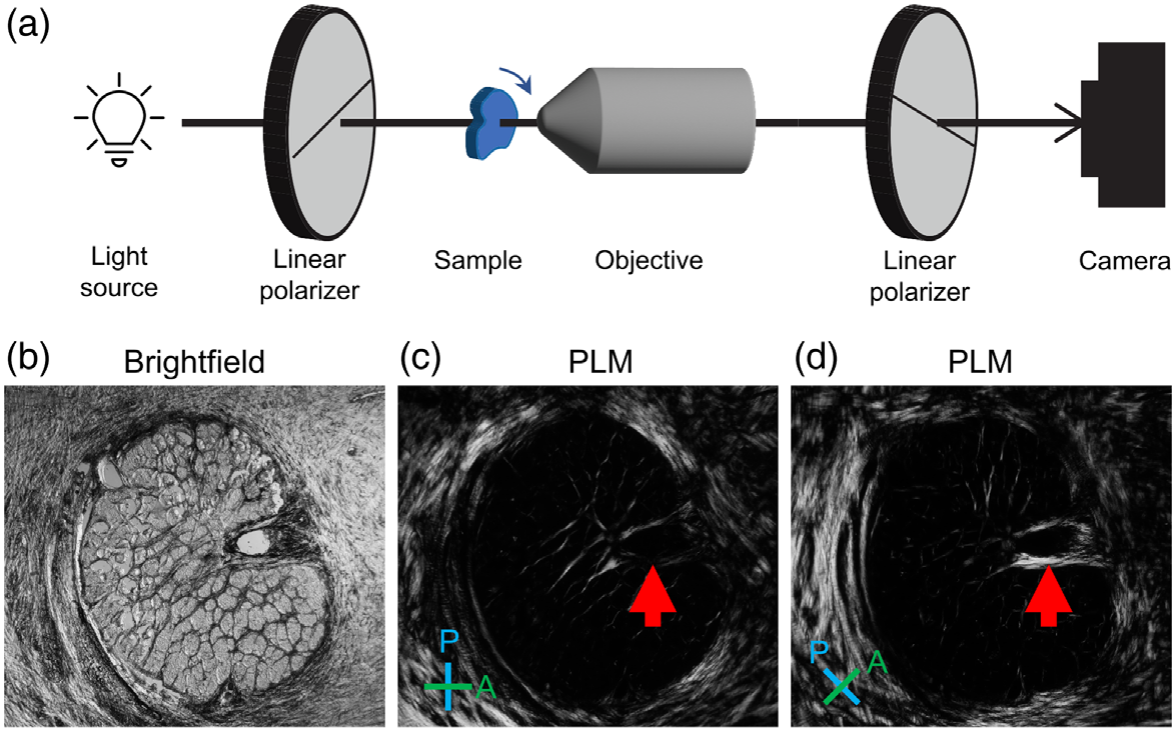
Cross-polarization schematic and example. (a) Optical scheme of cross-polarized light microscopy. (b)–(d) Collagen visualized with cross-polarization. A coronal cross-section of sheep optic nerve head, shown in brightfield illumination (b), and in two cross-polarized light setups (c), (d). The cross-polarized light setups are rotated 45 deg with respect to each other. Under the regular light of a brightfield microscope with no polarizers, it is difficult to distinguish collagen from other components of the tissue. Contrast is primarily from pigment absorption and scattering. Under crossed-polarized illumination, the collagen tissues appear brighter than the other tissue components due to their retardance. The maximum brightness of the collagen fibers at the same location (red arrows) varies when the relative orientation of the fibers and polarizer-analyzer setup (P, blue line and A, green line). For clarity, the two PLM images are shown in the same orientation, and it is the P-A setup that was rotated. It is these variations in brightness that are leveraged to quantify collagen orientation.

cPLM is the oldest and most basic form of PLM.^7^^,^^64^ The experimental setup for cPLM [Fig. 4(a)] utilizes a standard brightfield microscope and adds two optical components: a linear polarizer between the light source and the sample, and a second linear polarizer, or analyzer, somewhere after the sample and before the camera, typically after the objective. The two polarizers are oriented so that their transmission axes are perpendicular, called “cross-polarized.” In the absence of any other optical component or sample that will affect the polarization of light between the cross-polarized polarizers, any light that passes through the first polarizer will be polarized perpendicular to the transmission axis of the analyzer and will not be able to pass through to the camera. Therefore, no light will reach the camera in the absence of any birefringent material. If there is birefringent material present in the sample, and the optic axis of the sample is not parallel or perpendicular to the polarization of the incoming light, the polarization of the light will be rotated, and a portion of the light will then be able to pass through the analyzer and reach the camera. As light only reaches the camera in the case of a birefringent sample, the background for cPLM is black in the absence of any birefringent material. In addition, to ensure that all birefringent material is captured in an image, the cPLM image is typically repeated twice, once at an initial position of the two polarizers and sample, and once with both the polarizers^72^ or the sample rotated 45 deg from their initial position. This second image is measured with the polarizers rotated 45 deg so that any birefringent material that was parallel/perpendicular to the polarizers in the initial image and therefore not visible will be captured in the second image, as shown in Figs. 4(c) and 4(d).

The amount of light that can pass through the analyzer and reach the camera, or the brightness, is dependent on the in-plane orientation of the material and the phase retardance. For a given retardance, the in-plane orientation of maximum brightness is at 45 deg relative to the polarizers, which, for collagen fibers, is when the fiber axis is oriented 45 deg relative to the polarizers. In addition, if the fibers are oriented so that the fiber axis is at all out of the sample plane, this will also decrease the brightness. Fibers oriented at a small out-of-plane angle will appear brighter than those with a larger out-of-plane angle because, as the out-of-plane angle increases, a smaller component of the light is projected along the long fiber axis (extraordinary axis). Therefore, the same fiber appears darker when cut transversely.

cPLM is a great tool for visualizing if and where there is birefringent material, such as collagen fibers, present in a sample. It has even been used to obtain quantitative information on collagen fiber crimp.^73^ However, the method described here as cPLM is not great for quantifying the orientation of birefringent materials or quantifying the phase retardance. Next, we describe a very similar method for quantifying these two properties with PLM.

### 4.2 Quantitative Polarized Light Microscopy

**Fig. 5:**
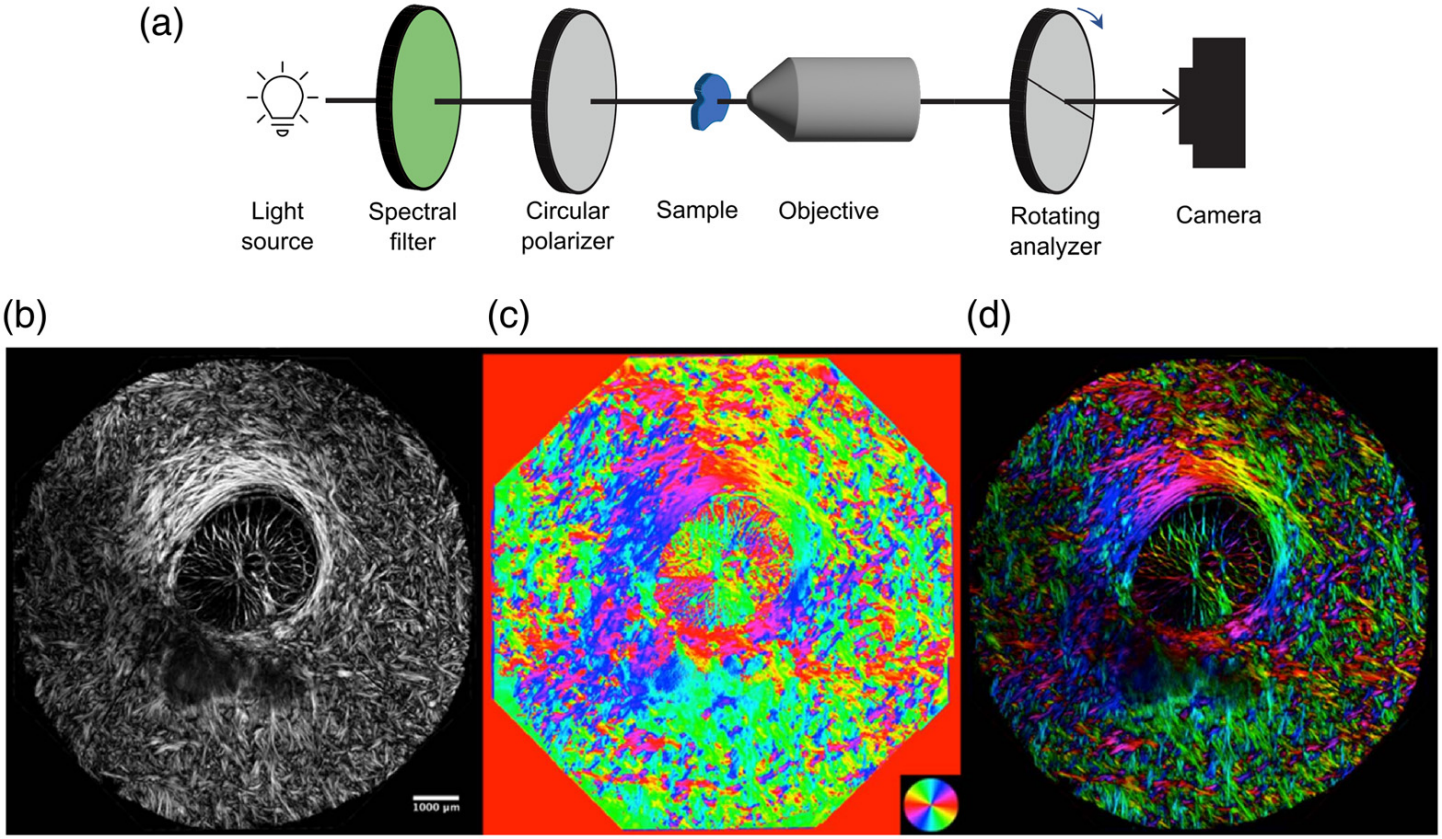
Quantitative polarized light microscopy schematic and example. (a) Optical scheme of quantitative polarized light microscopy. An optical filter is necessary because the circular polarizer is sensitive to the wavelength. (b)–(d) Images of coronal sections of sheep optic nerve head images with quantitative polarized light microscopy. Shown are (b) retardance magnitude, (c) orientation, and (d) a combination where the colors indicate the orientation and the brightness is weighted by retardance (adapted from with permission from Ref. 76).

Quantitative PLM extends the methods of cPLM to quantify local retardance and orientation of birefringent materials.^8^^,^^74^^,^^75^ PLM follows the same general process as cPLM: two polarizers are used, one before and one after the sample, to measure how much the polarization of light is rotated at each pixel. The major difference for PLM is that an optical filter and a circular polarizer replace the first linear polarizer from the cPLM scheme. The optical filter is necessary to narrow the bandwidth of light used in the experiment because the circular polarizer is sensitive to the wavelength of light. One PLM optical scheme utilizing this method is shown in Fig. 5(a). With a circular polarizer, light will be able to pass through the analyzer even in the absence of birefringent material, which leads to a gray background. Birefringent material alters the polarization of the light, leading to either an increase or a decrease in the amount of light passing through the analyzer. The amount of light that passes through the analyzer depends on the orientation of the extraordinary axis of the material, the long fiber axis for collagen, relative to the orientation of the analyzer. Multiple images are measured with the analyzer at various orientations. After the images are registered, polarization algorithms are used to calculate the local phase retardance and fiber orientation at each pixel in the image.^76^

One common algorithm utilizes four frames, with the analyzer set at 0, 45, 90, and 135 deg to capture the intensities $I_{0}$, $I_{45}$, $I_{90}$, and $I_{135}$, respectively. The in-plane orientation can then be calculated as^77^

$$\phi =\frac{1}{2}\;\tan^{-1}\;\frac{I_{90}-I_{0}}{I_{135}-I_{45}},$$ (11)

and the phase retardance can be calculated as

$$\delta =\sin^{-1}(\frac{2\sqrt{(I_{90}-I_{0}{)}^{2}+(I_{135}-I_{45}{)}^{2}}}{I_{0}+I_{45}+I_{90}+I_{135}}).$$ (12

In practice for visualization, the retardance is often represented by a separate quantity referred to as “energy.” The energy can be calculated as^8^

$${\text{energy}}^{2}=(I_{90}-I_{0}{)}^{2}+(I_{135}-I_{45}{)}^{2}.$$ (13)

**Fig. 6:**
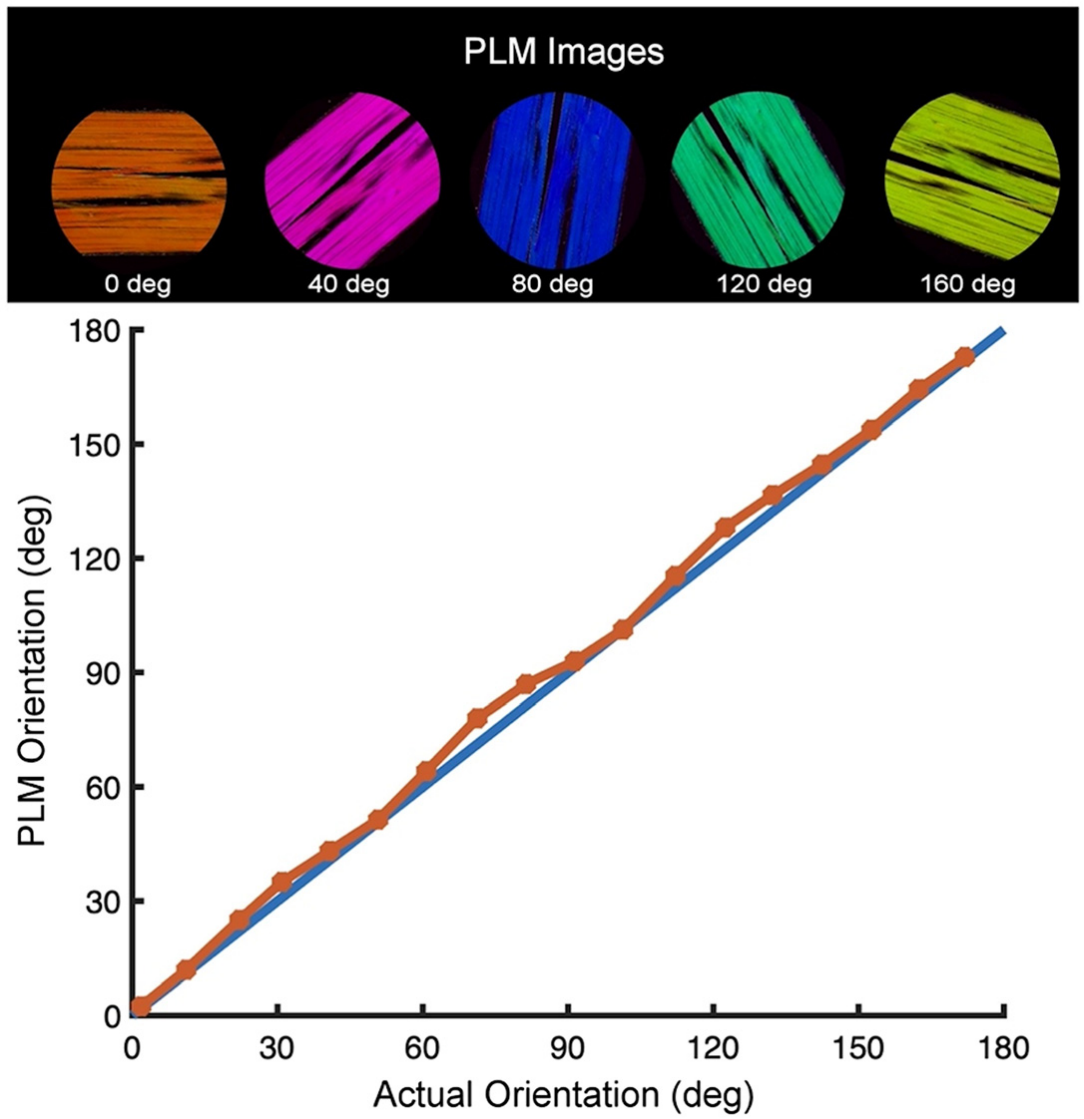
PLM images of chicken tendon at various set angles (actual orientation) are then used to calculate the orientation of collagen fibers (PLM orientation). The actual orientation and PLM orientation match extremely well, demonstrating the accuracy of PLM for determining fiber orientation.

In Fig. 6, we demonstrate the accuracy of this four-frame method and algorithm. Chicken tendon was sectioned and imaged with PLM. The collagen fiber microstructure is very anisotropic in tendon, with almost all the fibers completely aligned, and therefore, the calculated orientation should be the same for the whole sample. The tendon was then rotated in steps of 10 deg through 180 deg and the PLM image was collected at each orientation of the tendon. The actual orientation versus the orientation calculated from the PLM images is shown in Fig. 6. The relationship between the two is linear with only a small amount of error.

Not only is PLM accurate in its orientation determination, but it is also repeatable and has micrometer-scale resolution over a broad field of view. Some of PLM applications have been to characterize the collagen microarchitecture in the basal cochlear turn^4^ and optic nerve head [Figs. 5(b)–5(d)],^76^ microstructural remodeling of articular cartilage,^6^^,^^78^ bone mechanical function,^79^ collagen deposition in burn healing,^80^ wall structural integrity of brain arteries,^81^ and paths of white matter tracts in the brain.^5^

**Fig. 7:**
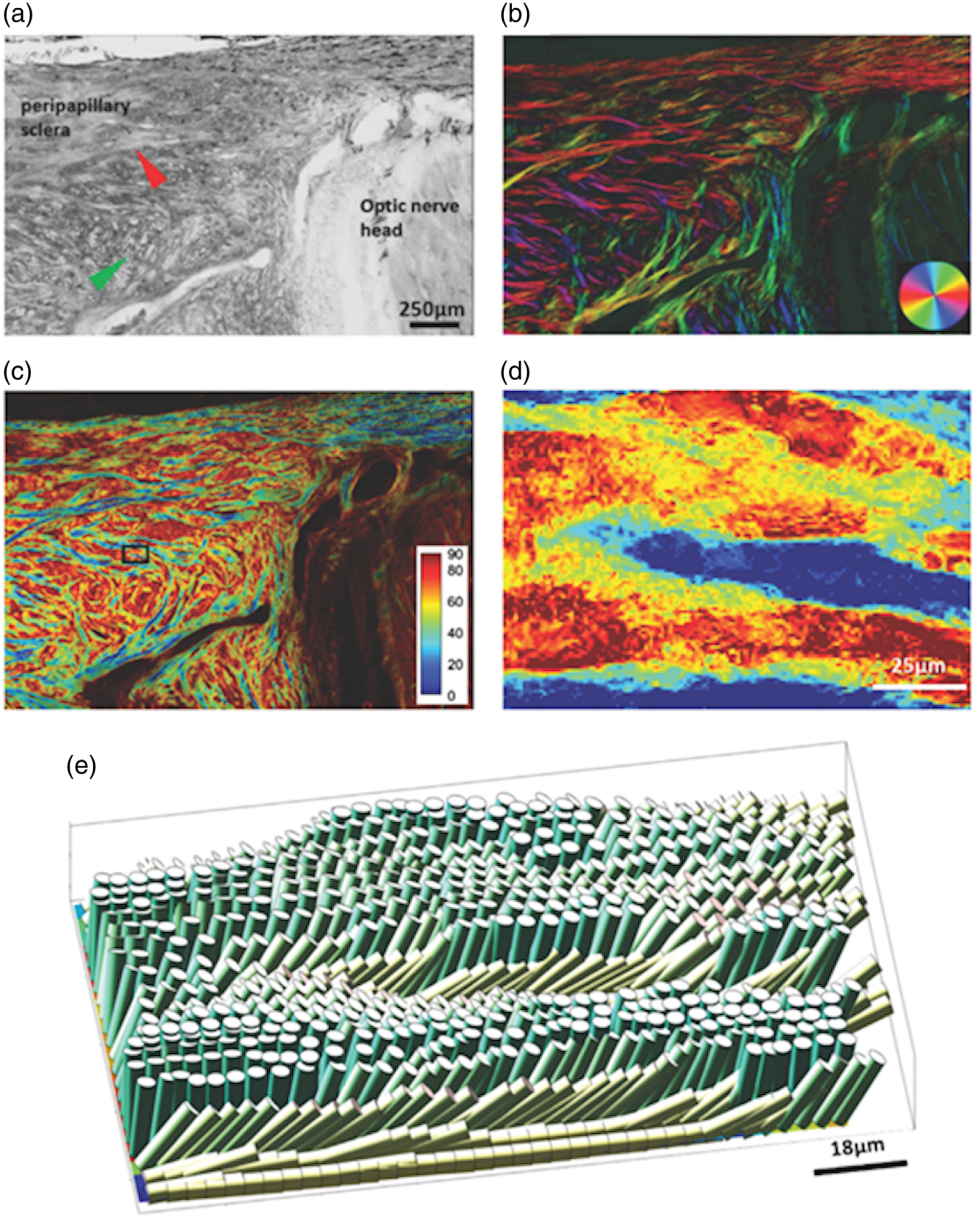
Quantitative polarized light microscopy for three-dimensional mapping of collagen fiber architecture in the posterior pole of a sheep eye. (a) Bright-field image with red and orange arrowheads pointing to long in-plane fiber bundles and out-of-plane fiber bundle fascicles, respectively. (b) In-plane fiber orientation map weighted by energy showing both in-plane fiber morphology and orientation. (c) Out-of-plane fiber orientation map highlighting fiber bundle fascicles. (d) Out-of-plane fiber orientation of small ROI shown in panel (c). (e) 3D visualization of collagen fibers (reprinted with permission from Ref. 69).

As mentioned for cPLM, the brightness decreases for greater out-of-plane angles. For PLM, this corresponds to a smaller measured retardance when the extraordinary (long fiber axis) is directed out of the plane. However, the out-of-plane orientation can be estimated if it is assumed that there is little variation in the retardance across the birefringent material (Fig. 7). This estimation can be done using Eq. (10) from the above theory section.^69^ The reference retardance is determined by the maximum retardance (brightness) in the image. The assumption of near constant retardance is an assumption that the birefringence and thickness have little variation across the sample. Another assumption made in this estimation is that the fiber dispersion is low as the variation in effective retardance also increases with fiber dispersion. Therefore, out-of-plane orientation estimations are best made for thin tissue sections imaged with microscale resolution but must be cautiously considered in areas of high fiber dispersion. The out-of-plane orientation can also be determined by changing the angle of incidence of the light with the sample.^82^

**Fig. 8:**
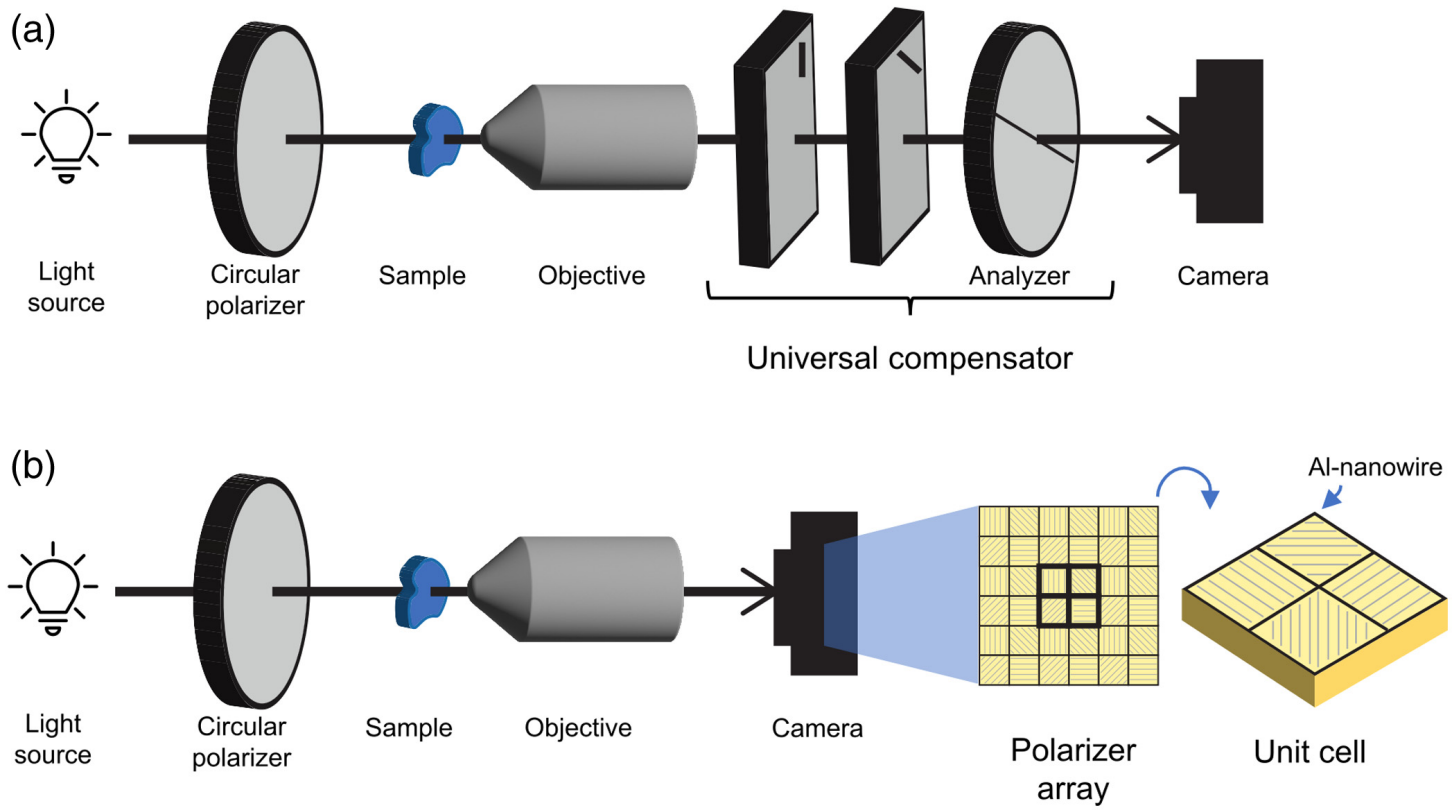
Two alternative schemes of quantitative PLM. (a) Optical scheme of LC-PolScope: two variable liquid crystal retarders are used to replace the rotating polarizer in traditional quantitative PLM. (b) A rotating analyzer is not necessary when a CMOS image sensor constructed with polarization sensitivity is used, where aluminum nanowires placed directly on top of photodiodes act as linear polarization filters with four polarization orientations at 0, 45, 90, and 135 deg.

The optical scheme in Fig. 5(a) shows just one way of measuring PLM images. This scheme requires physical and typically manual rotation of the analyzer to collect the necessary polarization states for quantitative assessment of the retardance and orientation. Over the years, additional PLM schemes have been developed that fully automate the acquisition of the various polarization states necessary for PLM. Some schemes simply use computer control to switch/rotate the analyzer directly.^74^ Other schemes add in a compensator to generate the desired polarization state without the need to change the analyzer. Traditional compensators have a variable optic path length that is controlled by rotation or electro-optic modulation via Pockels cells^83^ and Faraday rotators.^84^ A universal compensator can also be used, which is made of two liquid crystal variable retarders and a linear polarizer.^85^^–^^87^ Any arbitrary polarization state can be set with a universal compensator by changing the voltage applied to each liquid crystal retarder. This scheme is demonstrated in Fig. 8(a). Another method for automated PLM imaging removes the need for a separate analyzer altogether; either the imaging beam is split,^88^^,^^89^ or the camera itself is polarization sensitive,^90^^,^^91^ as shown in Fig. 8(b). Via nanofabrication techniques, a polarization-sensitive coating can be applied to the photosensitive elements (e.g., CMOS image sensor). One example would be to monolithically integrate aluminum nanowires on the photosensitive element at four orientations (0, 45, 90, and 135 deg) arranged in a 2-by-2 grid. Each orientation of aluminum nanowires acts as a polarization filter. Identical to how the retardance and orientation can be determined for the intensity of the images for the analyzer at the four different angles, the signal from each of the four pixels in the 2-by-2 grid can be used to determine the retardance and orientation from the same equations. The polarization-sensitive detector eliminates the need for multiple images to calculate the orientation and retardance, which allows for real-time image acquisition.^92^ In addition, cameras with nanowire filters are now commercially available (e.g., DZK 33UX250, The Imaging Source, Bremen, Germany), whereas customization is required for other forms of implementing PLM.

### 4.3 Instant Polarized Light Microscopy

A recent advancement in PLM called IPOL utilizes white light, the wavelength dependence of the real part of the refractive index of quartz, and a color camera to determine the orientation and phase retardance with a single frame. IPOL is based on techniques first introduced in 2015 by Shribak,^93^ and its experimental and quantitative methods were further detailed by Yang et al. in 2021.^41^ Via post-processing, it is possible to quantitatively determine the orientation of collagen fibers and their retardance from IPOL images, and it is also possible to visualize in a single snapshot the fiber dispersion qualitatively without any post-processing.

**Fig. 9:**
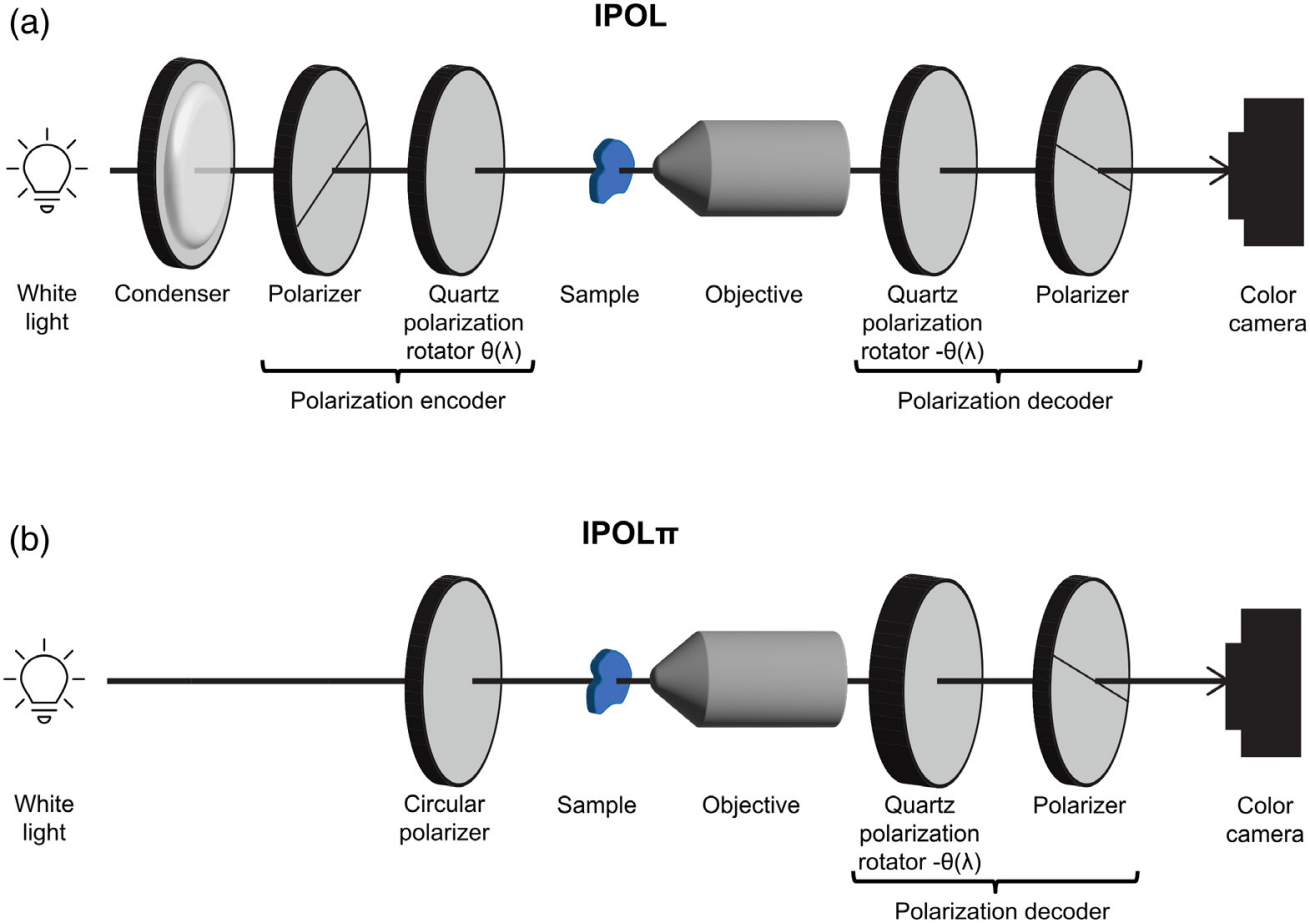
Optical scheme of instant polarized light microscopy (a) and IPOLπ (b). A condenser is necessary to collimate the light into a parallel beam because polarization rotators are sensitive to the optical path.

**Fig. 10:**
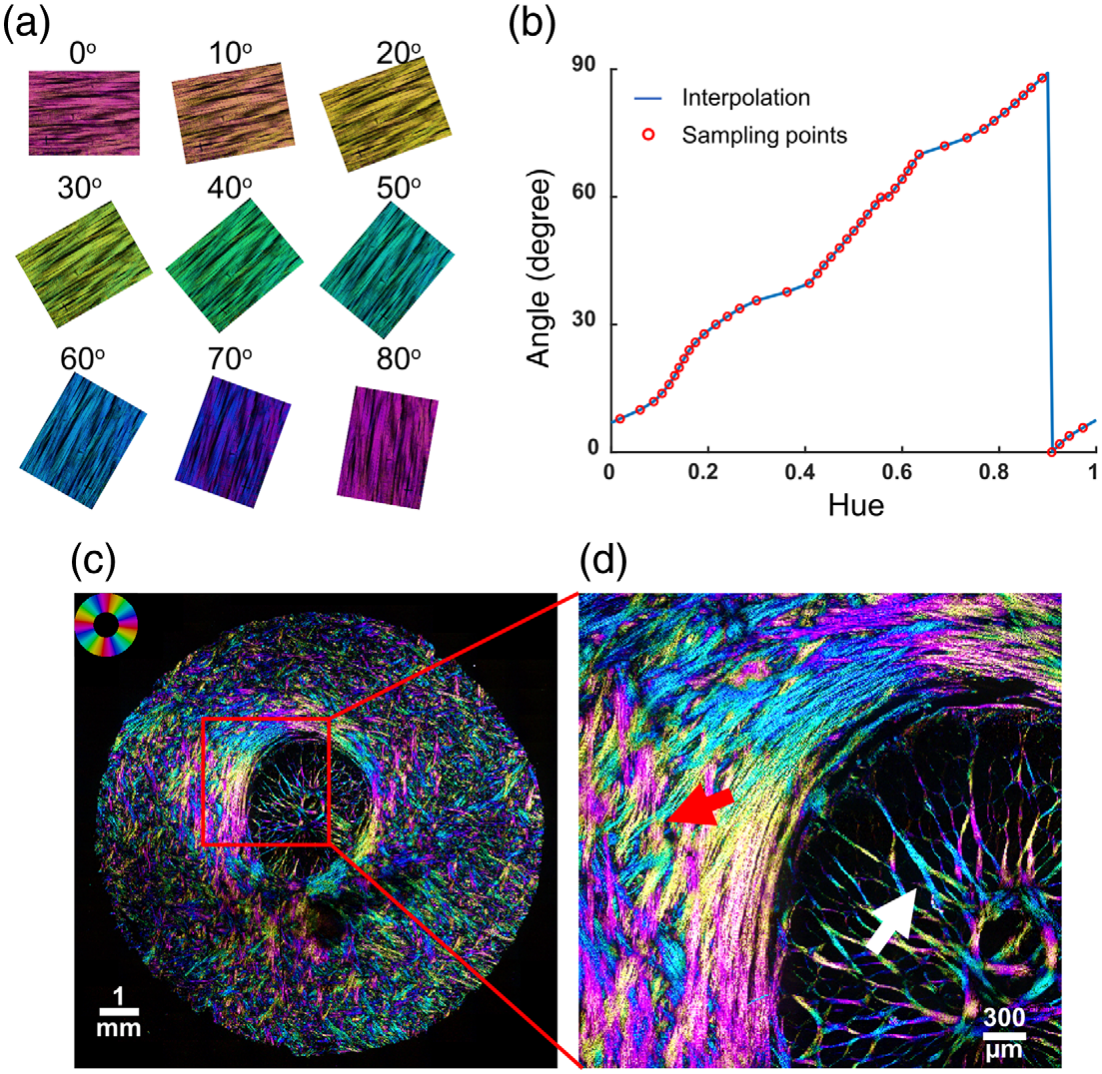
Example calibration of IPOL. (a) Raw IPOL images of chicken tendon sections at different orientations, where hue is correlated with the fiber orientation. (b) Circular interpolation of fiber orientation as a function of hue. There is a monotonic relationship between the hue and fiber orientation within 90 deg. (adapted with permission from Ref. 41). (c) Raw IPOL image of a sheep optic nerve head coronal section. Close-up of optic nerve head revealed highly detailed collagen fiber features: crossing fiber bundles (red arrow) and collagen fiber undulations or crimp (white arrow). The color wheel indicates the correspondence between pixel color and fiber orientation.

The optical scheme for IPOL requires three additional components compared with the simplest PLM experiments [Fig. 9(a)]. The optical scheme is composed of a condenser after the white light source, a polarization encoder before the sample, and a polarization decoder after the sample. The light is then detected with a color camera. The polarization encoder and decoder are made of a linear polarizer and polarization rotator (z-cut quartz). The polarizers in the encoder and decoder are cross-polarized, and the polarization rotators are opposite-handed. The polarization encoder first linearly polarizes the light independent of the wavelength, then the z-cut quartz in the encoder rotates the polarization of light, but the angle of rotation is dependent on the wavelength of light, θ(λ), diverging within 90 deg. As the z-cut quartz in the decoder is opposite handed to the quartz in the encoder, the light is rotated the same amount in the opposite direction. According to Mueller calculus, the effective polarizing element of the z-cut quartz pair and the sample, S, can be expressed as

$$M_{\mathrm{ROT}}(-\theta (\lambda ))\cdot S\cdot M_{\mathrm{ROT}}(\theta (\lambda ))=S_{\mathrm{ROT}}(\theta (\lambda )),$$ (14)

where the sample can be viewed as a waveplate. Thus, if there is no birefringent sample to rotate the polarization at the sample, the light will return to the same linear polarization as the encoding linear polarizer and will not be able to pass through the cross-polarized decoder polarizer. This results in a dark background for IPOL images, making IPOL suitable for epi-illumination. In IPOL, each wavelength of the light source has its own polarization after the encoder, such as measuring a cPLM image at many different orientations of cross-polarized linear polarizers. Therefore, the color of the light that can pass through the decoder indicates the orientation of the collagen fiber at a given pixel. The IPOL image is equivalent to a superimposition of many PLM images, where the superimposition is a spectrum that can be measured with a color camera or a spectrometer. When detecting with a color camera so that only the RGB values are measured, the hue of the color image varies with the fiber orientation within 90 deg [Fig. 10(a)], and the brightness is approximately linearly proportional to the retardance.

Aside from the fiber orientation, there are three factors that affect the color observed in IPOL: the white light source, the microscope system, and the absorption profile of the tissue. First, the spectrum of the white light can vary greatly depending on the fluorescent lamp or LED being used. The bandwidth of the light source must be broad enough that after passing through the z-cut quartz, all angles 0 to 90 deg are accessed. Second, the microscope system can induce spectral aberration or attenuation due to the optics and the coatings used on the optics. There may be aspects of the system or tissue that decrease light transmission, such as pigment in the tissue absorbing light. Naturally, if all the light is absorbed, an angle cannot be calculated at that pixel and will appear black in the IPOL image. To obtain rigorous quantitative information from IPOL images that are consistent across various imaging systems, an experimental calibration is needed for each IPOL imaging system [Fig. 10(b)].^25^^,^^41^ The sample used for calibration should be highly uniform in its fiber orientation, such as a tendon. The calibration process can control for the spectrum of the white light and any spectral aberration/attenuation from the imaging system but not for any absorption from the tissue. The use of a microscope-specific calibration means that IPOL imaging is not limited in any additional ways by the microscope system compared with traditional brightfield microscopy: IPOL can achieve the same spatial resolution and image quality. The ability to resolve different angles in IPOL is set by the light source and the thickness of z-cut quartz.

**Fig. 11:**
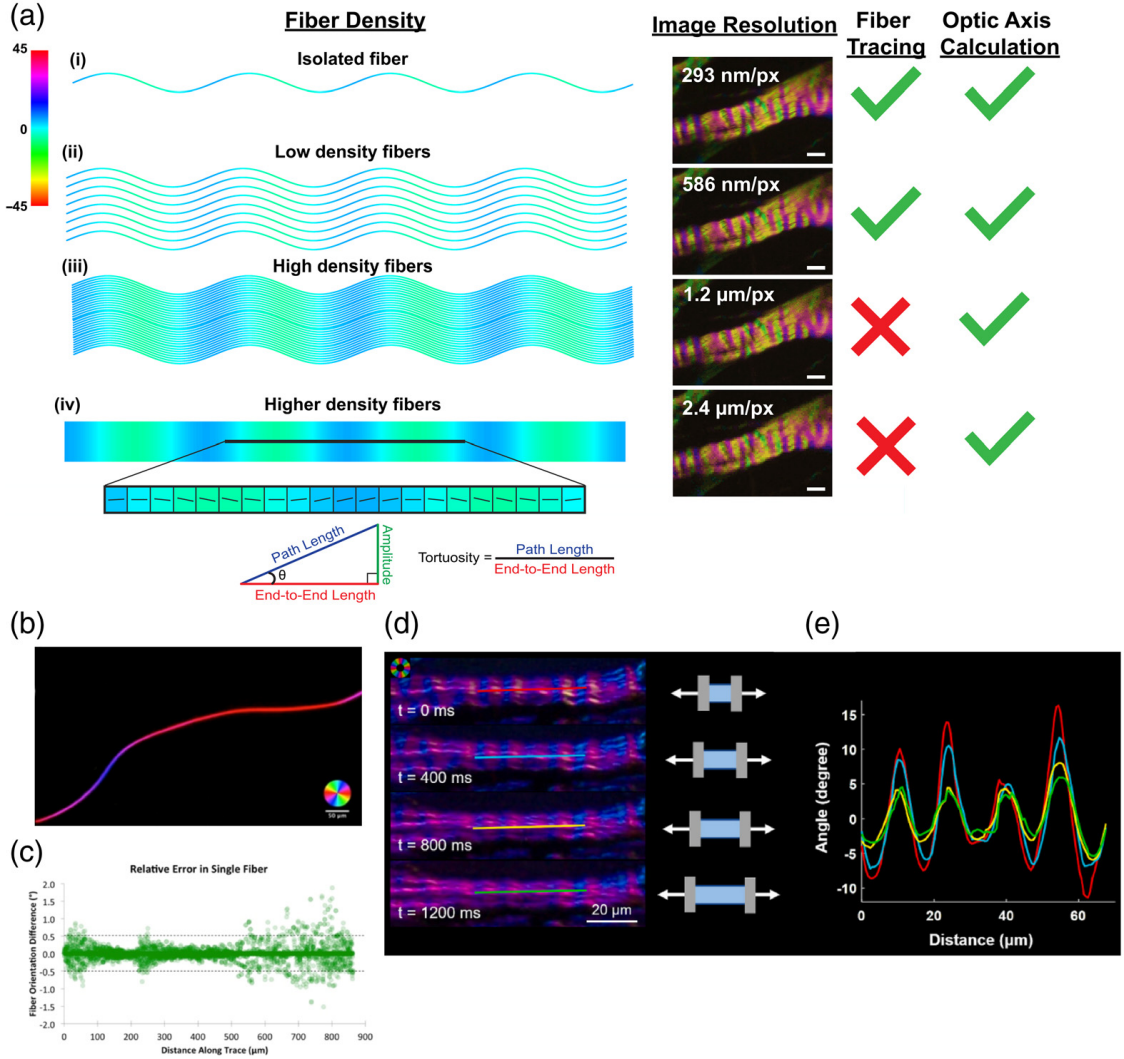
Traditional methods of calculating collagen fiber orientation and properties of crimp require the ability to trace fibers by visualizing the edges of fibers. This is possible in situations of isolated fibers (i), and high-resolution images with a low enough density of fibers to still distinguish fiber edges.^94^ In polarization methods, the orientation of the fibers corresponds to a particular color (color bar, −45  deg to 45 deg). In the situation of either low resolution and/or a high density of fibers (iii), fiber edges cannot be distinguished, but the color corresponding to fiber orientation is still known. Using the orientation (theta) at each pixel and the pixel size (end-to-end length), the path length, amplitude, and tortuosity of the collagen crimp can be calculated for each pixel and ROI. Previously, a single silk thread (b) was imaged with PLM. The fiber orientation was calculated based on manual tracking and Eq. 11 (c), demonstrating the ability of polarized light methods to recreate the traditional fiber tracing methods.^8^ An example from IPOL (d), (e) shows the color bands of crimp along a sheep lamina cribrosa beam. The fiber orientation along the ROI is plotted as the beam is stretched, even though individual fibers cannot be traced.^25^

Similar to PLM, IPOL encodes the local fiber orientation and retardance in each image pixel, but unlike PLM, these features can be obtained in a single snapshot and viewed qualitatively without any post-processing. Therefore, IPOL can preserve the full spatial and temporal resolution of the imaging system. In addition, like PLM, IPOL is a great tool for visualizing collagen microarchitecture, such as collagen crimp and interweaving, but the imaging speed of IPOL is limited only by the speed of the camera, which makes it ideal for imaging complex tissue deformation under load, like viscoelasticity.^25^ Another aspect of IPOL that distinguishes it from PLM is that the hue detected is cyclic every 90 deg. As the whole spectrum is cycled through in just 90 deg, this provides fine resolution for fiber orientation to observe features such as fiber crimp. A major advantage of all polarization methods, demonstrated in Fig. 11 with IPOL, is the ability to quantify various crimp parameters without having to physically trace fibers or visualize fiber boundaries.^25^^,^^48^^–^^50^

An unfortunate side effect of the hue cycling every 90 deg is that perpendicular fibers are indistinguishable by hue alone. One way to circumvent this ambiguity is to use texture-based Fourier analysis; another way is a further advancement in polarization microscopy called IPOLπ.^95^

**Fig. 12:**
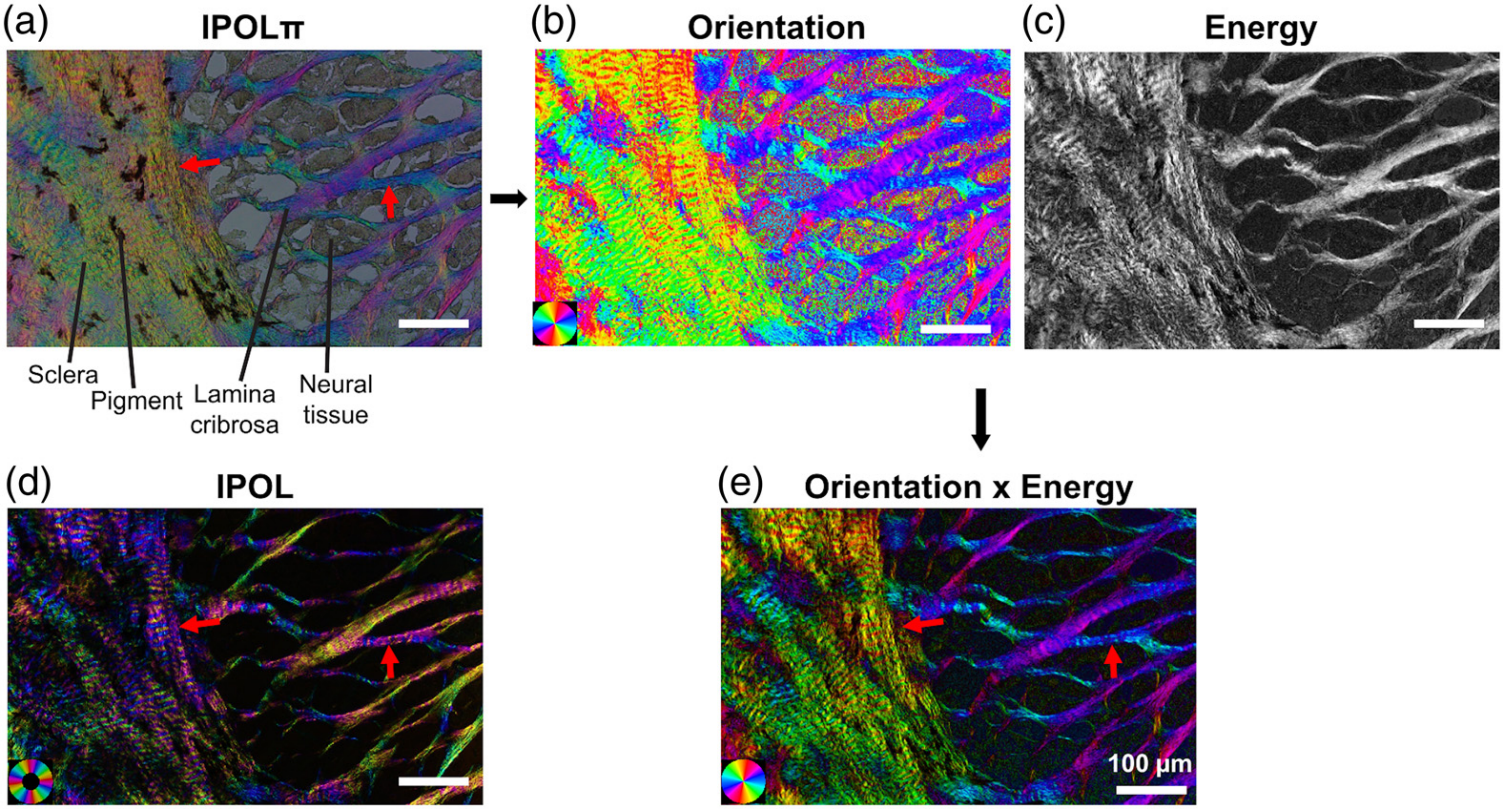
Comparison of IPOL and IPOLπ of the same sample. Panel (a) shows a raw IPOLπ image of the sheep optic nerve head (i.e., as acquired). The collagen fibers of the sclera and lamina cribrosa are seen in color. Between the lamina cribrosa beams, the neural tissue can be seen, and pigment can be visualized among the scleral collagen fibers due to the gray background in IPOLπ, in contrast to the IPOL image in panel (d). The IPOL image has a black background, and therefore, only the collagen can be seen. The raw IPOLπ image can be used to quantitatively determine the orientation (b) and energy (c). Panel (e) shows the orientation weighted by the brightness determined from IPOLπ. Comparing the IPOL image and the processed IPOLπ image in panel (e), the red arrows correspond to perpendicular collagen fibers. In the IPOL image, the fibers are the same color, but the IPOLπ color can distinguish the two orientations. Image adapted with permission from Ref. 95.

IPOLπ further simplifies the optical scheme of IPOL by replacing the polarization encoder with a circular polarizer [Fig. 9(b)]. Like when a circular polarizer is used for PLM imaging, the sample is simultaneously interrogated by a linear combination of all linear polarizations at once; however, different from PLM, in IPOLπ, all the relative orientations of the analyzer are read out simultaneously with the white light illumination source and the polarization decoder. The z-cut quartz in the decoder separates the colors into different elliptical polarizations, and then, the linear polarizer permits a portion of the elliptically polarized light to pass through. In the absence of any birefringent material, all the colors can pass through the decoder polarizer equally, yielding a gray background [Fig. 12(a)]. If there is birefringent material present, certain colors of light will be able to pass through the polarizer more or less than the background, leading to a color image. The colors that can pass through are dependent on the orientation of the birefringent material. Like PLM and IPOL, the effect of the various polarization optics can be predicted via Mueller calculus. Two advantages of IPOLπ over are, first, because a circular polarizer is used instead of a linear polarizer before the sample, IPOLπ can display the collagen fiber orientation in color that is cyclic every 180 deg, making it possible to distinguish perpendicular fibers, and second, the gray background allows simultaneous visualization of nonbirefringent material. One added difficulty of IPOLπ versus IPOL is that in IPOLπ, there is not a direct proportionality between the hue and the phase retardance; instead, a more sophisticated RGB colormap is necessary to determine the retardance from the hue.^95^

### 4.4 Structure Polarized Light Microscopy

All the previous methods described here have relied on the transmission of light through tissue, limiting the methods to thinly cut sections of tissue tens of micrometers thick and preventing imaging in three dimensions. The reliance on transmitted light also prevents the previous methods from being used for imaging *in vivo* or *in situ* dynamics such as pressure-induced deformations. Although there have been many beautiful experiments done with PLM or IPOL and sectioned tissue, it is difficult to reconstruct the 3D architecture from sectioned tissue, and the sectioning itself is time-consuming and destructive to the tissue. SPLM, developed in 2018 by Yang et al., combines structured light illumination and PLM and collects the reflected light for thick specimen imaging.^96^

**Fig. 13:**
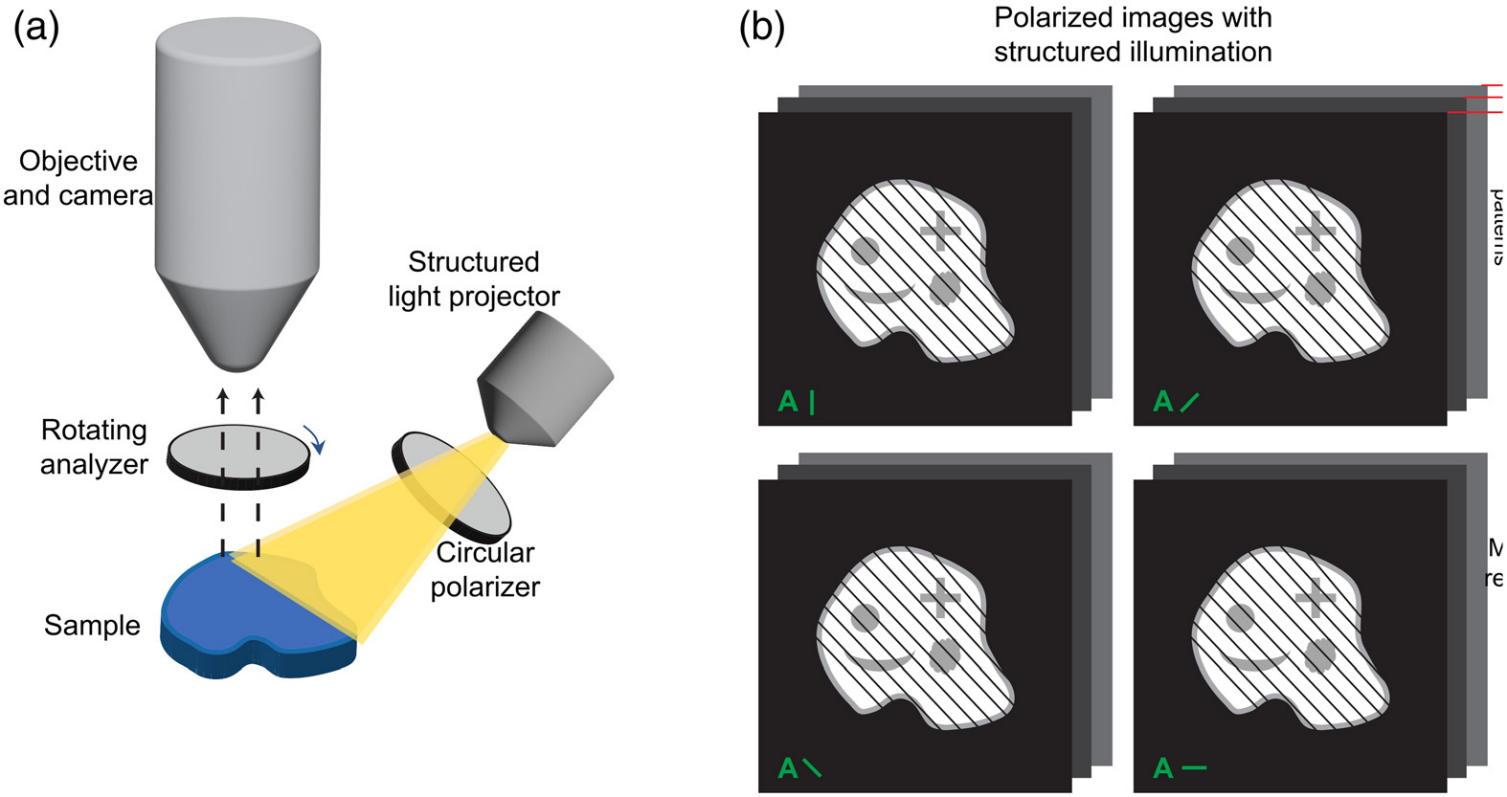
(a) Schematic illustrating the steps involved in obtaining an optically sectioned quantitative image using SPLM. 12 images are required to create an SPLM image: four polarization conditions (b) and three phase-shifted images at each polarization. Note that off-axis illumination helps avoid strong reflected light.

The optical components for SPLM are very similar to PLM, except that the light source is replaced with a structured light projector (Fig. 13). As in PLM, a circular polarizer is placed after the light source and before the sample, and a rotating analyzer is placed between the sample and objective. One difficulty of using reflected light for imaging is the interference of diffuse light from out-of-focus planes with the in-focus imaging plane. This diffuse background makes it difficult to measure small changes in intensity. Structured light illumination eliminates the diffuse background through a combination of optical and computational methods.^97^ For SPLM, a fringe pattern is projected onto the sample through a physical mask or with a spatial light modulator, such as a digital micro-mirror device (DMD). A set of images at the desired polarization states, such as the four used for PLM, is collected. A set of images at the four polarization states are also collected for two additional fringe patterns, phase-shifted 120 and 240 deg. The intensity for each polarization state, such as $I_{0}$, $I_{45}$, $I_{90}$, and $I_{135}$, is computed from the intensities of the three fringe patterns: $I_{1}$, $I_{2}$, and $I_{3}$. The structured light illumination intensity for the polarization state, $I_{\mathrm{SLI}}$, is then^98^

$$I_{\mathrm{SLI}}=\frac{\sqrt{2}}{3}\sqrt{(I_{1}-I_{2}{)}^{2}+(I_{2}-I_{3}{)}^{2}+(I_{3}-I_{1}{)}^{2}}.$$ (15)

Once the intensity for each polarization state has been computed from the three fringe patterns, the orientation and retardance can be computed from the same equations used for PLM. The imaging depth of SPLM is determined by the frequency of the fringe pattern, and thus, optical sectioning can be done by changing the frequency.

**Fig. 14:**
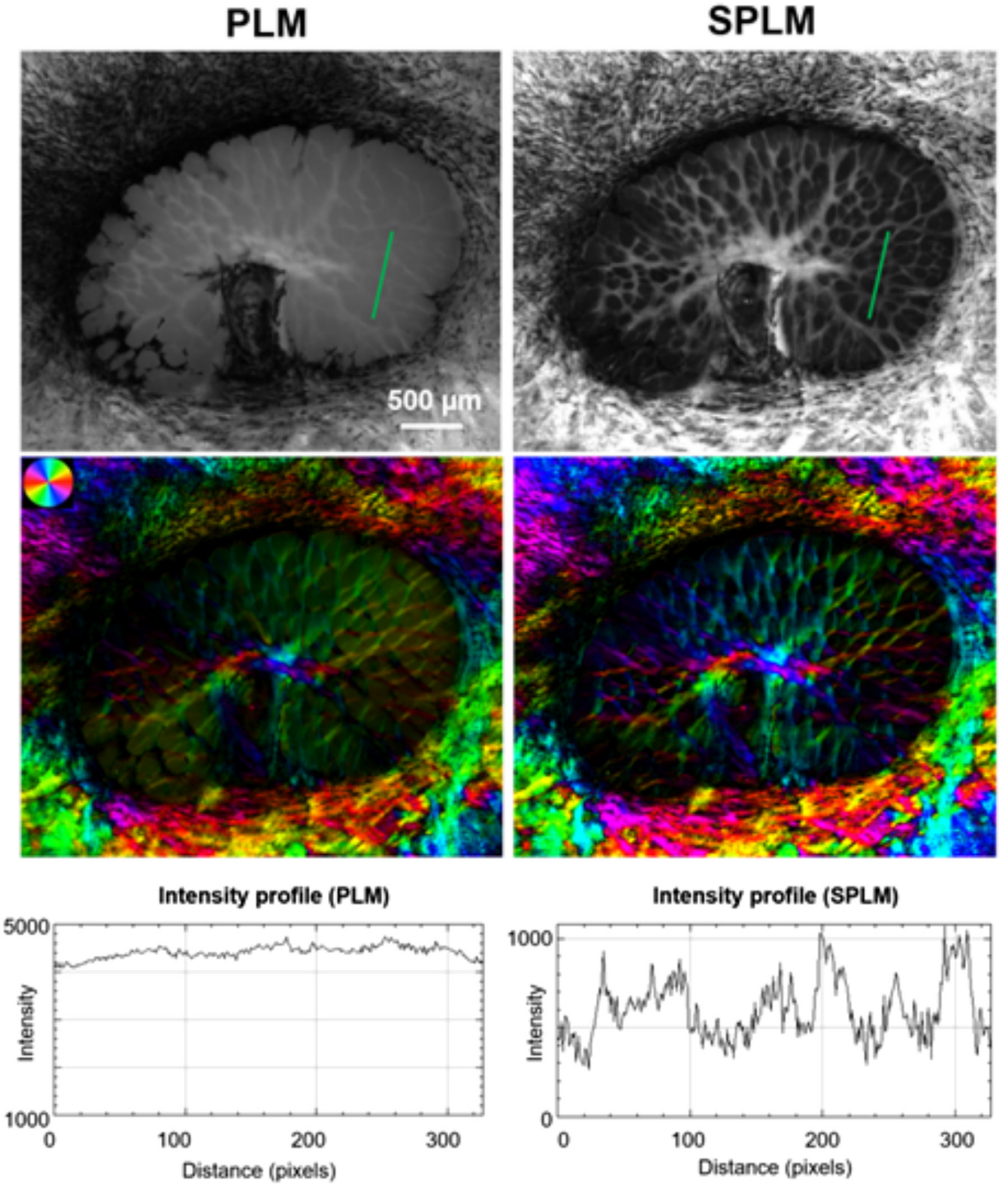
PLM and SPLM comparison of an *in situ* sheep optic nerve head. The SPLM image shows greater contrast between the collagen fibers and the surrounding tissue (adapted with permission from Ref. 96).

A demonstration of the benefits of SPLM versus PLM is shown in Fig. 14. PLM images of thick tissues measured via reflected light show very little contrast between collagen fibers in the optic nerve head of the sheep eye. SPLM eliminates the diffuse background to visualize more detail and creates fiber orientation maps that are more accurate. The development of SPLM enables polarization microscopy of thick tissue undergoing mechanical testing, which can help build a link between the microarchitecture and the macroscale mechanical properties of tissue.

### 4.5 Polarization Sensitive-Optical Coherence Tomography

**Fig. 15:**
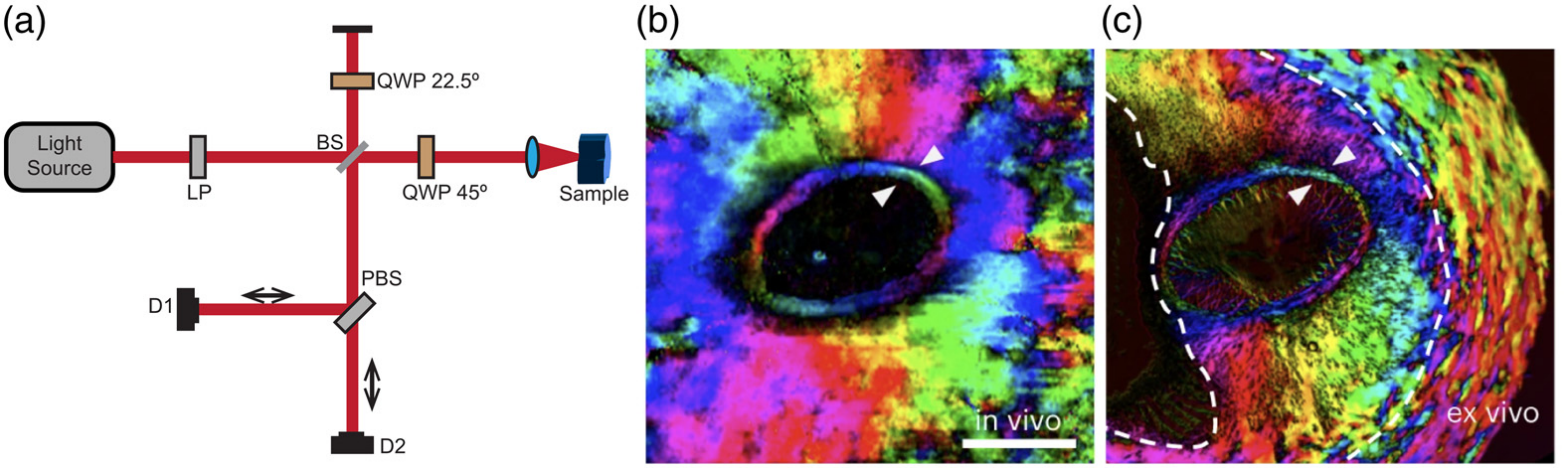
PS-OCT single input state diagram (a). LP, linear polarizer; BS, beam splitter; QWP, quarter-wave plate; PBS, polarization beam splitter; D, detector. Pig optic nerve head imaged *in vivo* with triple-input PS-OCT (b) and *ex vivo* with PLM (c). Scale bar 1 mm. Panels (b) and (c) were adapted with permission from Ref. 17.

PS-OCT is another imaging modality that measures tissue birefringence and optic axis orientation. PS-OCT measures the polarization dependence of the OCT signal (i.e., spectral interferogram) through coherent detection. OCT is based on low-coherence light interferometry,^99^ which uses a low-coherence light source and an interferometer, as depicted in Fig. 15(a). The light from the source is split into a reference arm and a sample arm with a beam splitter. The light in the sample arm reflects off some scatterer in the sample and is then recombined with the light in the reference arm and sent to a detector. The light from the sample arm will interfere with the light in the reference arm destructively or constructively, depending on the optical pathlength difference (OPLD) between the two. In OCT, the light reflected off the sample at different depths is recombined and interfered with the reference light, which encodes their OPLD information into a spectral interferogram. By either changing the length of the reference arm (time-domain OCT) or detecting the spectral interferogram with a spectrometer (spectral-domain OCT; SD-OCT) or measuring it with a swept laser source that sweeps its instantaneous wavelength over a wide bandwidth (swept-source OCT; SS-OCT), OCT can retrieve the OPLD information of the sample at different depths and reconstruct the depth-resolved reflectance profile of the sample (the A-scan or axial scan) from the spectral interferogram. These A-scans are compiled to create a B scan, which is compiled into a volume image. Further theoretical and experimental details of traditional OCT can be found in various reviews.^100^^–^^102^ An advantage of OCT imaging is its wider adoption for *in vivo* imaging in ophthalmology, dermatology, cardiology, oncology, and dentistry,^102^^,^^103^ as it does not rely on transmitted light such as PLM and IPOL. In addition, OCT uses coherent detection and thus is more sensitive to singly-scattered than to multiply-scattered light than SPLM and yields the degree of polarization (DOP) equal to 1 (i.e., $I^{2}=Q^{2}+U^{2}+V^{2}$). Depending on the wavelength of the light source and the scattering/absorption spectra of the tissue, OCT image depths can reach 8 to 10 mm. It has also been implemented in endoscopes for imaging inside the body.^104^^,^^105^

PS-OCT methods range from simple, a single polarized input state,^106^^–^^108^ to complex, multiple inputs to obtain all 16 elements of the tissue Müller matrix.^17^^,^^109^ Here, we will describe only the simplest form of PS-OCT methods to understand the principle, and a more detailed description can be found in various reviews.^15^^,^^66^^,^^110^ Single input PS-OCT (Fig. 15) starts with a linear polarizer after the light source so that the light entering the reference and sample arms has the same linearly polarized light. Using Jones formalism, we can define a vertically polarized light from the source as a normalized Jones vector: $E_{\mathrm{in}}=[\begin{array}{c} 0\\ 1 \end{array}]e^{-ikz}$, where $e^{-ikz}$ is a reduced exponential propagator used later for describing the coherent detection. In the sample arm, after the beam splitter is a quarter-wave plate (QWP) oriented at 45 deg, which outputs circularly polarized light; $E_{\mathrm{circ}}=\frac{1}{\surd 2}J_{\mathrm{QWP}(45\;\deg)}[\begin{array}{c} 0\\ 1 \end{array}]e^{-ikz}$, where $J_{\mathrm{QWP}(45\;\deg)}=\frac{1}{\sqrt{2}}[\begin{array}{cc} 1 & i\\ i & 1 \end{array}]$. The circularly polarized light is used here for the same reason it is used in PLM. With circularly polarized light, it will capture almost all linear birefringence except the one whose optic axis is aligned parallel to the imaging beam. For brevity, here, we assume the birefringent tissue is a single linear retarder, with Jones matrix that is $J_{S}(\phi ,\delta )=R(\phi )\Lambda (\delta )R(-\phi )$, where $\Lambda (\delta )=[\begin{array}{cc} e^{i\delta /2} & 0\\ 0 & e^{-i\delta /2} \end{array}]$ is an orthogonal phase retardation matrix with retardance δ and $R(\phi )=[\begin{array}{cc} \cos\;\phi & -\sin\;\phi \\ \sin\;\phi & \cos\;\phi \end{array}]$ is a rotation matrix with the orientation ϕ. After the light transmits the birefringent tissue and reflects off, it passes through the same birefringent tissue, QWP, and beam splitter again in a backward manner. Thus, the light returned from the sample arm can be described as,

$$[\begin{array}{c} H_{\text{sample}}\\ V_{\text{sample}} \end{array}]=\frac{1}{4}{J_{\mathrm{QWP}45\;\deg}}^{T}J_{S}^{T}\sqrt{R_{\text{sample}}}J_{S}J_{\mathrm{QWP}45\;\deg}[\begin{array}{c} 0\\ 1 \end{array}]e^{-ikz}\;=\frac{\sqrt{R_{\text{sample}}}}{4}{J_{\mathrm{QWP}45\;\deg}}^{T}J_{S}(\phi ,2\delta )J_{\mathrm{QWP}45\;\deg}[\begin{array}{c} 0\\ 1 \end{array}]e^{-ikz}\;=\frac{\sqrt{R_{\text{sample}}}}{4}[\begin{array}{c} \cos\;\delta \cdot e^{-\delta i}\\ \sin\;\delta \cdot e^{i(\pi -\delta -2\phi )} \end{array}]e^{-ikz},$$ (16)

where $J_{S}(\phi ,2\delta )=[J_{S}(\phi ,\delta ){]}^{T}J_{S}(\phi ,\delta )$ is the round-trip Jones matrix of the sample and $R_{\text{sample}}$ is the sample reflectance of the birefringent tissue and divided by four because the light passes the beam splitter twice, $H_{\text{sample}}$ is the horizontal polarization component, and $V_{\text{sample}}$ is the vertical polarization component. If there were no birefringent tissue (δ=0), the light returning from the sample arm to the beamsplitter would once again be linearly polarized and rotated by 90 deg, but if there is birefringent tissue, the light would be elliptically polarized, with an axis ratio that is determined by the retardance δ and phase difference by the optic axis orientation ϕ. Note that the sample reflectance is hypothetically polarization independent and measured at the given depth after the light has passed through the birefringent tissue.

In the reference arm, the light passes through another QWP but at 22.5 deg so that after double passing through the QWP the reference arm light is linearly polarized at 45 deg, namely, $[\begin{array}{c} H_{\text{reference}}\\ V_{\text{reference}} \end{array}]=\frac{\sqrt{R_{\text{mirror}}}}{2\sqrt{2}}[\begin{array}{c} 1\\ -1 \end{array}]e^{-ikz_{0}}$, where $z_{0}$ is determined by the round-trip travel distance in the reference arm and becomes a constant in the case of SD/SS-OCT. After the light from the reference and sample arms is recombined, a polarizing beam splitter splits the light into 0 deg (horizontal) and 90 deg (vertical) polarization components, which are detected by each detector,

$$I_{H}(k)=|H_{\text{sample}}+H_{\text{reference}}{|}^{2}\;I_{V}(k)=|V_{\text{sample}}+V_{\text{reference}}{|}^{2},$$ (17)

Fourier transforming both Eq. (17) yields their respective cross-correlation terms that represent the depth profile of the PS-OCT signal,

$$A_{H}(z^{\prime })\propto \sqrt{R_{\text{mirror}}\cdot R_{\text{sample}}(z^{\prime })}\;\cos\;\delta (z^{\prime })\cdot e^{-\delta (z^{\prime })i}\cdot e^{-ikz^{\prime }}⊗\Gamma (z^{\prime })\;A_{V}(z^{\prime })\propto \sqrt{R_{\text{mirror}}\cdot R_{\text{sample}}(z^{\prime })}\;s\text{in }\delta (z^{\prime })\cdot e^{-(\pi -\delta (z^{\prime })-2\phi (z^{\prime }))i}\cdot e^{-ikz^{\prime }}⊗\Gamma (z^{\prime }),$$ (18)

where $z^{\prime }=(z-z_{0})/2$ is the half of OPLD between the reference and sample arm (which accounts for the round-trip travel distance), $\Gamma (z^{\prime })$ is the modulus of complex degree of coherence determined by the light source, which yields the depth resolution of OCT, and ⊗ is a convolution operator.

Using Eq. (18), the sample reflectance at a certain depth $z^{\prime }$ is obtained from the intensity (the amplitude squared) of the two polarization components combined:

$$R_{\text{sample}}(z^{\prime })\propto A_{H}(z^{\prime }{)}^{2}+A_{V}(z^{\prime }{)}^{2}.$$ (19)

The retardance at the depth $z^{\prime }$ can be obtained from the ratio of the vertical and horizontal amplitudes:

$$\delta (z^{\prime })=\tan^{-1}(\frac{A_{V}(z^{\prime })}{A_{H}(z^{\prime })}).$$ (20)

Last, the optic axis orientation at the depth $z^{\prime }$ can be determined from the phase difference between the vertical and horizontal components, $\Delta \Phi =∠[A_{H}(z^{\prime })\cdot A_{V}^{*}(z^{\prime })]$:

$$\phi (z^{\prime })=\frac{\pi -\Delta \Phi (z^{\prime })}{2}.$$ (21)

The values in Eqs. (19)–(21) are all derived from Jones formalism, but the same conclusion can be obtained from Stokes–Mueller matrix formalism using the fact that DOP = 1 for coherent detection. Hence, PS-OCT, unlike PLM, provides depth-dependent information. As Eqs. (7) and (13) show, the retardance depends on the amount of birefringent material the light passes through, so the measured PS-OCT retardance and optic axis are cumulative with the depth, regardless of the methods. It is noteworthy that, however, these “cumulative” retardance and optic axis properties can change nonlinearly (increase, decrease, or no change) depending on the combination of birefringent materials the light passes through. Therefore, we do not expect the measured retardance to linearly increase with the depth in thick tissue unless the sample is well-defined and is a well-aligned uniaxial birefringent thin-section tissue, which will be discussed later.

The greatest strengths of PS-OCT are its ability to image *in vivo* and collect 3D information. The *in vivo* aspect of PS-OCT makes it a potential tool for diagnosing various diseases.^18^^,^^111^^–^^116^ With the advantage of *in vivo* imaging also comes the challenges and constraints of working *in vivo*. One of the greatest challenges is obtaining high-resolution images as high-resolution imaging requires objectives with large numerical aperture (NA) and short working distances. For example, one of the most common clinical uses of OCT is to image the back of the eye,^117^ which limits the OCT device to a working distance of the diameter of the human eye (2.2 to 2.5 cm) and the pupil size available for imaging reduces with age, cataract, and other eye conditions. These constraints often limit the lateral resolution to 10s of μm, which can obscure much of the microarchitectural detail that can be obtained with PLM or IPOL. Implementation of adaptive optics (AO) can increase the lateral resolution of PS-OCT to 2.5  μm when imaging the retina and optic nerve head through a fully dilated pupil.^118^

However, the ability to extract 3D information of the microarchitecture is nontrivial due to the polarization state of the light interacting with the sample being depth dependent, which is because the tissue the light passes through is birefringent and its optic axis varies with the depth. Therefore, to compare the optic axis information at different depths quantitatively, the evaluation of the optic axis in each optical section must be done for each polarization element. In addition, even if the birefringent tissue preserves the same optic axis orientation across the depth, other factors, such as the impact of linear diattenuation and multiply-scattered light, increase as the depth increases, causing measurement errors. Recently, heuristic iterative approaches have been proposed for resolving a single polarization element in the thick tissue and/or mitigating some systematic errors.^19^^,^^119^^,^^120^ Further development, along with histological validation, is needed for accurately resolving a single polarization element *in vivo*.

**Fig. 16:**
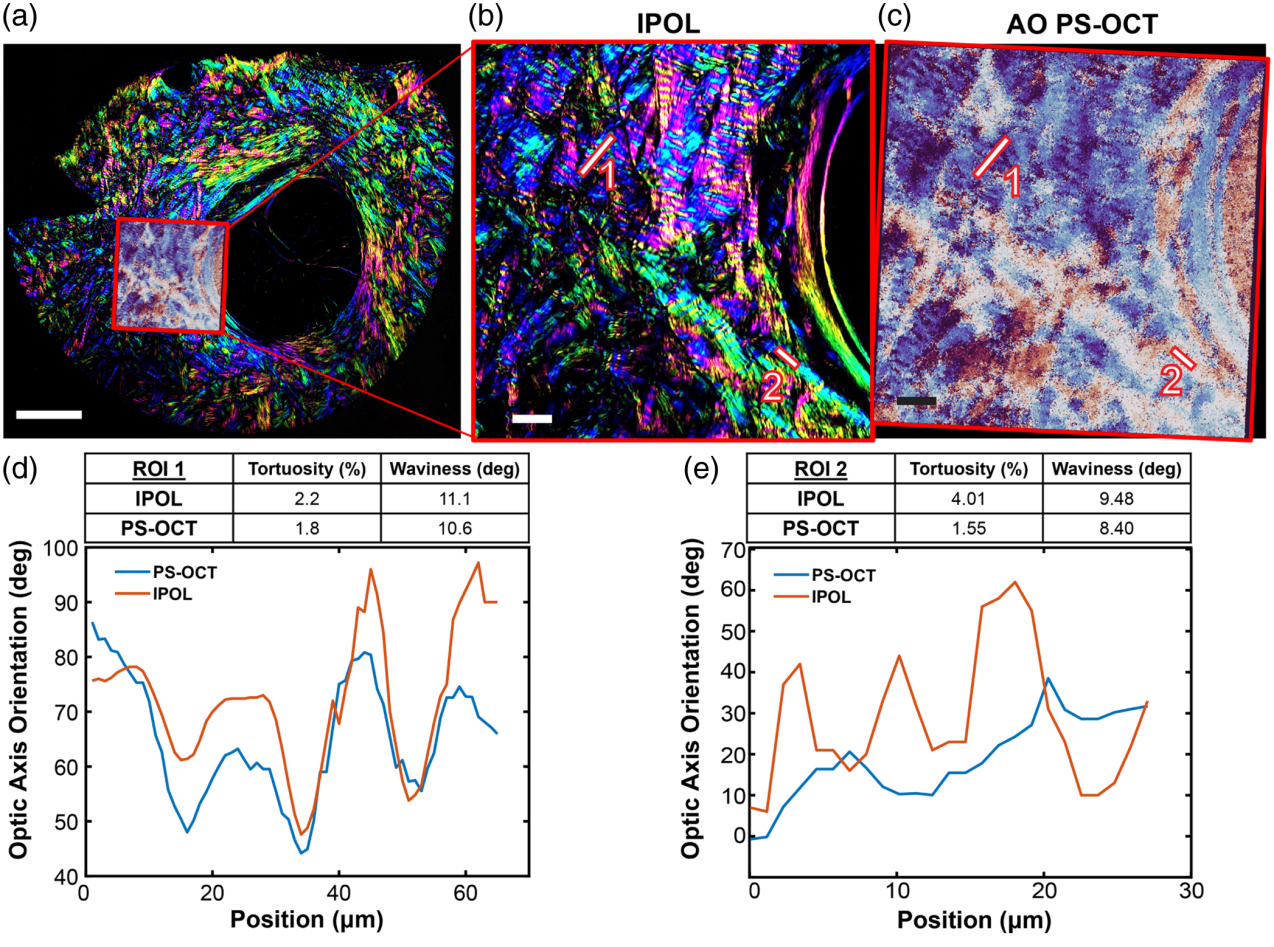
Comparison of IPOL and PS-OCT of the same sample. (a) Fixed sheep optic nerve head section 30  μm thick, imaged in entirety with IPOL, and a sclera region of interest near the canal imaged with AO PS-OCT overlayed. The region in the red box is shown imaged with both IPOL (b) and PS-OCT (c). Undulations in the collagen are visible in both methods. The angles along two ROIs (white lines 1 and 2) are plotted for both methods. The tortuosity and waviness are calculated for both methods. The two methods match well for ROI 1, farther from the canal (d), but PS-OCT does not measure any crimp for ROI 2 next to the canal (e). The scale bars denote 500  μm in panel (a); 50  μm in panels (b) and (c).

Indeed, such a “development and validation” attempt has already begun at the laboratory level (Drs. Sigal and Kurokawa labs). Figure 16 shows an initial result that PS-OCT can measure the same crimp patterns as measured by IPOL using a single tissue section from a sheep eye. We made a one-to-one comparison of the crimp waviness in sclera measured by IPOL and AO PS-OCT (but with a long focal length f=75  mm (NA = 0.05), emulating low-resolution research/clinical instruments). Farther away from the canal (region of interest 1; ROI 1), IPOL and PS-OCT are already in good agreement. However, in the sclera closest to the canal (ROI 2), PSOCT does not capture the collagen crimp that is measured with IPOL. We have previously shown that, using PLM, the period of the crimp is shorter closer to the canal, as is true when comparing ROI 1 and ROI 2.^50^ Thus, in its current state, PS-OCT is not capable of resolving crimp with periods of the length found in the peripapillary sclera closest to the canal and in the lamina cribrosa. This initial result underscores that further development and validation, especially under well-controlled perturbations, such as biaxial stretches and relaxations, will inform requirements for PS-OCT to determine the difference in disease, such as glaucoma. The current state of PS-OCT methods is powerful as this comparison shows that PS-OCT is capable of accurate crimp measurements of moderate period length when compared with a gold standard IPOL measurement. Last, this comparison shows PS-OCT combined with AO has great potential to image fine microarchitectural detail, such as crimp, *in vivo*.

With that said, the most challenging aspect of PS-OCT is its complexity in hardware and software implementation. Special care must be taken for system design, calibration, and data analysis. For example, the above-mentioned single-input PS-OCT has assumed perfect polarization optics with no wavelength dependency for theoretical formulation; in practice, it is impossible to make a perfect circularly or linearly polarized light across wavelengths using off-the-shelf components, which ultimately causes systematic measurement errors.^21^^,^^121^ Therefore, it is crucial to design and calibrate bulk optics, including their optical coating, such that both wavelength and polarization dependencies can be minimized. The same factors are relevant for fiber optics, even worse with mechanical and thermal disturbances, as these will impact the polarization state.^16^^,^^104^^,^^122^ Several PS-OCT system designs (e.g., dual-, triple-, and multiple inputs or single-input quad-detection Jones matrix PS-OCT) and advanced signal processing methods have been proposed, though these mitigation methods come at the expense of imaging depth, resolution, sensitivity, or speed. These extreme requirements make it harder to build a new PS-OCT system than a standard OCT system. Nonetheless, we anticipate more applications of PS-OCT in the coming decades, especially *in vivo* applications in the field of biomechanics.

### 4.6 Polarization-Resolved Second-Harmonic Generation Microscopy

**Fig. 17:**
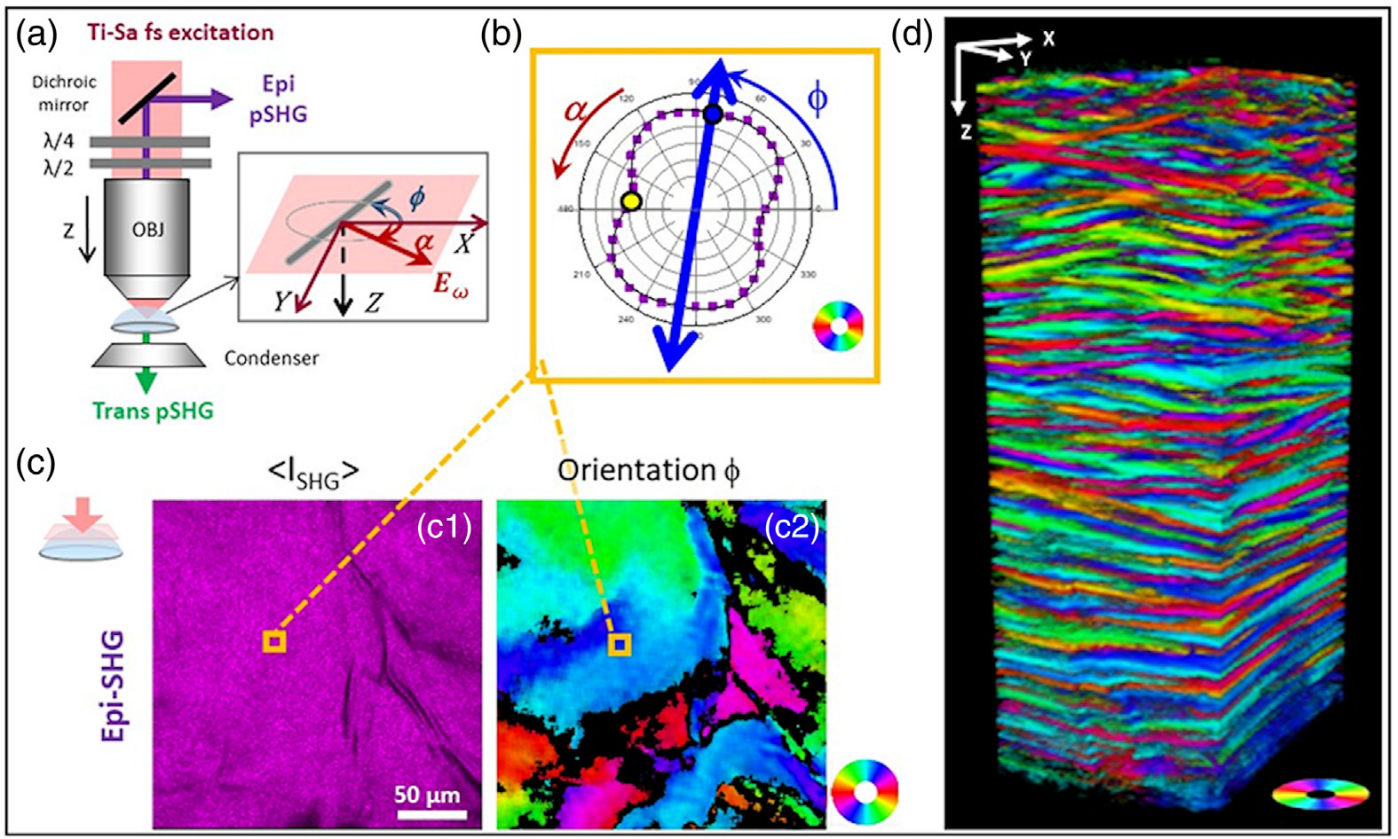
pSHG imaging of a whole human cornea. (a) Simplified schematic of a pSHG microscope showing the rotating waveplates used for polarization control at the back pupil of the objective lens. (b) pSHG polar plot measured in each voxel of the 3D image showing the SHG signal as a function of the orientation of the excitation polarization α; the main axis (blue double arrow corresponds to the collagen orientation ϕ and the square root of the ratio of the 2 minima (blue and yellow dots) provides a measure of the anisotropy parameter ρ. (c) En face images of a human cornea showing (c1) the average of all SHG images obtained for the series of excitation polarization orientations and (c2) the collagen orientation obtained using Eq. (19). (d) pSHG map of collagen orientation along the full thickness of a human cornea ($250\times 250\times 600\;{\mu m}^{3}$) showing the superposition of collagen lamellae with different orientations. Orientations are coded according to the color wheels in the insets. Adapted with permission from Ref. 125 (CC-BY 4.0 license).

3D information about the collagen structure at the micrometer scale can be obtained using pSHG microscopy. SHG is a coherent nonlinear optical process that appears at half the excitation wavelength. This signal can be detected in parallel with two-photon excited fluorescence (2PEF) in a multiphoton microscope using appropriate spectral filters. 3D images are then obtained by recording stacks of 2D images, thanks to laser scanning in the imaging plane and axial displacement of the objective. It allows 3D imaging of collagen in thick tissues with unprecedented sensitivity and specificity and without any labeling.^123^^,^^124^ pSHG consists of recording sequential SHG images with a series of linearly polarized excitations at different orientations [Fig. 17(a)]. The SHG signal in each pixel then depends on α−ϕ, that is, the orientation α of the excitation polarization with respect to the orientation ϕ of the collagen fibrils within the focal volume [Fig. 17(b)]:

$$I_{\mathrm{SHG}}(\alpha )=CI_{\mathrm{exc}}^{2}[(\rho \;\cos^{2}(\alpha -\phi )+\sin^{2}(\alpha -\phi ){)}^{2}+(\sin\;2(\alpha -\phi ){)}^{2}],$$ (22)

where $I_{\mathrm{exc}}$ is the intensity of the excitation laser and C is a combination of geometrical and other parameters. Fitting or FFT analysis of Eq. (22) then provides the orientation ϕ of the collagen fibrils within the imaging plane, which corresponds to the main axis of the pSHG polar plot [blue double arrow in Fig. 17(b)], and the so-called anisotropy parameter ρ, which corresponds to the square root of the ratio of the SHG signal for incident polarization parallel to the collagen fibrils to that for perpendicular polarization [blue and yellow dots at the two minima in Fig. 17(b)]. This can be automated numerically to provide 3D maps of collagen orientation and ρ values [Fig. 17(c)].

pSHG thus combines the orientation sensitivity of polarimetric techniques with the optical sectioning capability and the specificity for fibrillar collagen of SHG microscopy. It has been used by several groups to map the collagen structure in label-free tissues.^10^^–^^12^^,^^14^^,^^125^^–^^132^ It is easily combined with 2PEF microscopy using appropriate spectral filters because 2PEF signals are red-shifted compared with SHG. This is a key advantage because endogenous 2PEF signals show the tissue morphology.

SHG imaging can be done in the forward or backward directions, i.e., using epi- or trans-detection [Fig. 17(a)]. Epi-detection is convenient for thick and opaque samples and exhibits a smaller coherent length (∼100  nm), which allows better resolution of intricate collagen structures.^125^ Trans-detection allows analysis of the SHG signal along two orthogonal polarizations and thus provides more information on the collagen hierarchical structure than the basic implementation of pSHG with no analysis.^128^^,^^129^ More advanced pSHG implementation providing a generalized Mueller matrix has also been demonstrated in thin tissue sections. ^133^

pSHG advantageously provides a high-resolution image with typically 0.3  μm lateral resolution and 1.2  μm axial resolution (full width at half maximum) over a field of view of typically $500\times 500\;\mu m^{2}$. As a result of this strong focusing regime with a high NA objective, the polarization is distorted at the focus and comprises axial components. This effect affects the measurement of the anisotropy parameter ρ, but not the collagen orientation.^134^ The collagen orientation measured by pSHG is the one in the focal volume and is not affected by the cumulative birefringence in depth, in contrast to PS-OCT. In practice, the tissue birefringence distorts the pSHG polar plot in depth without rotating its main axis so that it affects the measurement of the anisotropy parameter ρ but not the collagen orientation.^14^ pSHG 3D mapping of collagen orientation is thus very robust and efficient [Fig. 17(d)].

pSHG also allows the measurement of the anisotropy parameter ρ, which probes the distribution of fibrils within the focal volume. It is equal to 1.36 for an assembly of fibrils perfectly aligned together in the imaging plane, which is related to the helical pitch of the collagen triple helix, and it increases with the orientational disorder or the out-of-imaging-plane orientation.^127^^,^^129^^,^^135^^–^^137^ This parameter can also be used to discriminate collagen from other protein assemblies that exhibit SHG signals, myosin, and tubulin. ρ is ∼1.3 to 2 for collagen (depending on the assembly), close to 0.5 for myosin and larger than 2 for tubulin.^138^^,^^139^

pSHG has several limitations. First, as a laser scanning imaging technique, it is slow compared with PLM. This limitation can be partially addressed in several ways, in particular by implementing fast polarization switching using an electro-optical modulator and by synchronizing the polarization switching with the line scan to achieve line acquisition times of a few tens of milliseconds.^140^^–^^142^ Second, it is an expensive technique compared with linear polarimetric techniques because it requires a femtosecond laser as the excitation source in the multiphoton microscope. However, collagen SHG is a nonresonant process that can be excited in a wide spectral range, so the advent of low-cost fixed-wavelength femtosecond lasers should reduce the cost of pSHG microscopy in the next decade.

Third, pSHG is based on linearly polarized excitation and probes the collagen orientation only in the imaging plane. However, orientation ψ out of the imaging plane can be accessed by reslicing 3D stacks in a transverse plane when the collagen density is low enough to isolate fibrils or fibers. In dense tissues or in 2D sections, out-of-plane collagen fibrils can be distinguished by their smaller SHG signal as $C\propto \cos^{2}\;\psi$ in Eq. (19) or by a larger anisotropy parameter ρ. These criteria are, however, ambiguous because SHG is a coherent process, which intensity is strongly dependent on the precise distribution of fibrils within the focal volume. A better method is to measure the normalized difference of SHG signals excited by left- and right-handed circular polarizations. This so-called circular dichroism-SHG (CD-SHG) vanishes for in-plane collagen fibrils and is nonzero only for out-of-plane fibrils.^143^^–^^146^ However, the sign and amplitude of the CD-SHG signal are not simply related to the sign of ψ but depends in a complex way on the polarity distribution of the out-of-plane fibrils.^146^^,^^147^

**Fig. 18:**
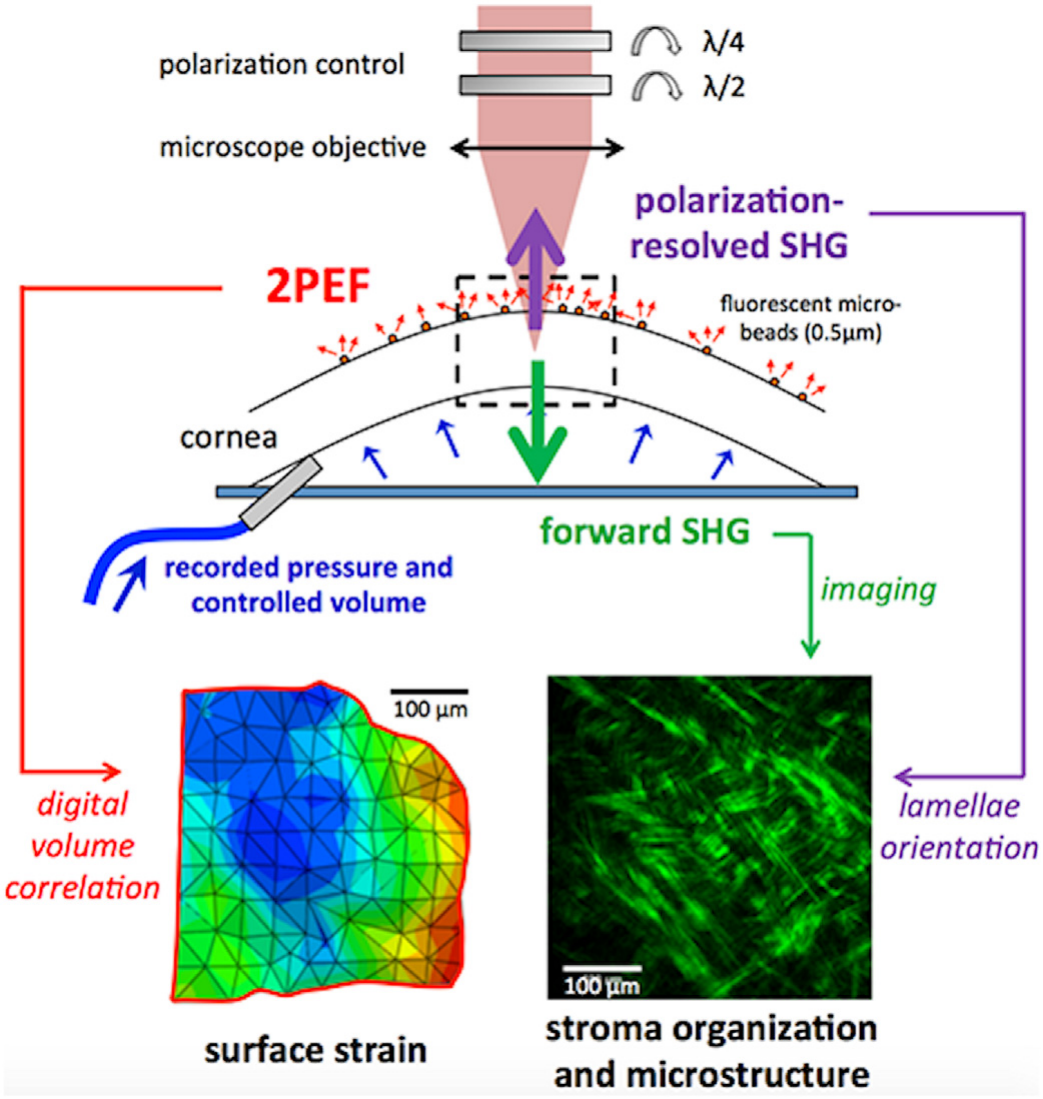
Schematic of inflation assays in *ex vivo* human corneas combined with pSHG imaging of the collagen lamellae in the stroma. (a) Rotating waveplates that control polarization at the back pupil of the objective lens; (b) cornea mounted on a chamber that enables controlled deformation (injected fluid volume) and pressure measurement. The colored arrows indicate detection of pSHG images in the backward direction (purple) and SHG images in the forward direction (green); 2PEF images of fluorescent microbeads on the cornea surface are also recorded in the backward direction. (c) A typical map of surface strain obtained by tracking fluorescent microbeads at different deformation steps using digital volume correlation. (d) A typical forward-detected SHG image of the corneal stroma. Adapted with permission from Ref. 148.

pSHG has been combined with mechanical assays by installing a traction or an inflation device under the objective.^136^^,^^142^^,^^148^ It allows multiscale characterization of the tissue mechanical properties by simultaneously recording macroscopic mechanical data (pressure for cornea, stress for tendon and skin) and micrometer-scale deformation of the collagen structure. In the cornea, for example, inflation assays mimic the increase of intra-ocular pressure during glaucoma (Fig. 18). They show a reorientation of the collagen lamellae with increasing pressure, which is correlated with the surface deformation along the same directions.^148^

## 5 Summary and Comparison of Polarization Method Major Strengths

PLM, IPOL(π), SPLM, PS-OCT, and pSHG are all methods capable of measuring collagen optic axis orientation and sample retardance. However, these methods each have different strengths.

PLM is a transmitted light method and, therefore, is best suited for thinner samples, such as tissue sections, cells, or other transparent tissue. The key advantage of PLM is its simplicity and low cost. PLM can be implemented in any bright-field microscope with the addition of one circular polarizer and one rotating linear polarizer.^8^^,^^74^ Determining the fiber orientation from Eq. (11) is an easy calculation and does not require microscope-specific calibrations.^77^ Because PLM relies on transmitted light, it cannot be used for *in vivo* measurements in mammals and cannot provide volumetric information. However, the out-of-plane fiber orientation can be recovered from PLM images with the assumption of little variation in retardance across the sample or using light inclination.^69^^,^^82^ Another strength of PLM is that it is a wide-field method that can be used in low or high magnifications (typically maxing out around 400 nm lateral resolution). In PLM’s simplest form, where all four polarization states are collected sequentially, the imaging speed is limited by how quickly the polarization states can be changed (often by a mechanically rotating element) and is often not suited for dynamic measurements. In versions of PLM where all four polarization states can be collected simultaneously or in quick succession, like with polarization-sensitive detectors, EOMs, or motorized polarizers, dynamic measurements such as stretching of tissue sections are possible.^92^

IPOL and IPOLπ are also transmitted light methods that are best suited for transparent tissue. IPOL and IPOLπ share many of the strengths of PLM, such as simplicity and spatial resolution. The key advantage of IPOL over PLM is that the fiber orientation can be calculated from a single camera frame, without the need for advanced electronics or detectors.^25^^,^^41^^,^^95^ An IPOL image can be collected in less than 10 ms and an IPOLπ image in less than 1 ms. These fast acquisition times make IPOL and IPOLπ the ideal techniques for imaging dynamic processes. Although faster than PLM, IPOL and IPOLπ require more expensive optics and later an involved conversion of image color to fiber orientation. Both IPOL and IPOLπ require windows of z-cut quartz in addition to the two polarizers as well as a color camera. For converting the color images to fiber orientation, a microscope-specific calibration is necessary.

When choosing between IPOL and IPOLπ, IPOL has a black background, and the angle-to-color calibration is cyclic every 90 deg, whereas IPOLπ can simultaneously visualize nonbirefringent material and is cyclic every 180 deg. However, an advantage of IPOL over IPOLπ is that the correlation between angle and hue is roughly linear for IPOL, making the conversion from color to angle relatively straightforward. The relationship between color and angle for IPOLπ is not linear, and therefore, the calculation of the angle from the color image is more involved. IPOL is also sensitive to light alignment between the two quartz filters, potentially causing artifacts in the periphery at low magnifications.

SPLM employs the same experimental components as PLM, but with the addition of a structured light projector as the light source.^96^ The collection of the PLM images at three different structured light phases enables PLM imaging in thick tissue, with the possibility of imaging *in vivo*. The need for three-phase images at each of the four polarization states means that SPLM is a slower method than PLM, but the computation of the fiber orientation is still a simple computation from Eqs. (11) and (15). The lateral resolution of SPLM is the same as that of PLM, but due to the slower acquisition time, measurements of dynamic processes cannot be done with a continuous measurement. An advantage of SPLM is that the in-plane fiber orientation can be measured at different depths by changing the frequency of the fringe pattern in the structured light.

pSHG is a point-scanning method capable of high-resolution (typically <300  nm lateral and 1.2  μm axial resolutions (FWHM) of <1  μm) volumetric imaging. SHG can image at maximum depths of 100s of μm in thick tissues, in both reflection and transmission modes. Therefore, it is an efficient method well suited for collecting 3D orientation maps in thick tissue sections or *ex vivo*. pSHG provides in-plane orientation of every voxel in depth using a simple calculation based on FFT.^134^ Out-of-plane oriented fibers can be highlighted using CD-SHG, and out-of-plane orientation can be estimated in transverse reconstructions.^143^^,^^146^ A key advantage of pSHG is its ability to specifically detect a few proteins—collagen, myosin, and tubulin—without the need for labeling, resulting in a high signal-to-noise ratio compared with other techniques. Furthermore, pSHG can distinguish between these three proteins, producing highly specific orientation maps. Another advantage is that pSHG can easily be combined with 2PEF, enabling the simultaneous imaging of the tissue morphology. Although SHG microscopy is a complex and expensive technique, multiphoton microscopes can be purchased commercially, and pSHG can be done with the addition of a single polarization component. With the development of resonant scanning mirrors, the entire field of view for a single optical section can be measured at a single polarization state in a few milliseconds; however, many optical sections are needed to create an image volume, and many polarization states are necessary to calculate the fiber orientation, making dynamic measurements with pSHG difficult. Nevertheless, using an electro-optical modulator to implement fast polarization switching and synchronize it with the line scan enables line acquisition times of a few tens of milliseconds.^142^ Although this makes dynamic measurements, such as stretching tissue sections, possible, it is still less optimal than IPOL and IPOLπ.

PS-OCT, which is a functional extension of OCT, is best suited for *in vivo*, noninvasive, and volumetric imaging applications. PS-OCT shares the major strengths of OCT. For example, OCT is capable of maximum imaging depths of hundreds of micrometers to many millimeters, with <5  μm axial resolution. The NA for OCT is typically less than 0.1, yielding a lateral resolution of 10 s of μm. The NA is often limited by the physical constraints of *in vivo* measurement, such as working distance, pupil size, and aberrations. Notably, the imaging speed of OCT has improved in the past decades, which is extremely important for *in vivo* applications. Current commercially available OCTs can capture a single depth scan within a few microseconds, but research-grade OCTs can acquire a single depth scan within a microsecond. PS-OCT is an ideal technique for longitudinal tracking of disease development and progression, both in the clinic^112^^,^^114^ and in animal models.^111^

## 6 Other Techniques

The methods summarized here cover approaches ideal for tissue sections (PLM and IPOL), 3D high-resolution imaging (pSHG), and *in vivo* measurement (PS-OCT) of collagen architecture. Another polarized light imaging method is Müller matrix polarimetric microscopy (MMP). MMP is most likely a PLM experiment but uses additional polarization conditions to extract the full Müller matrix of the sample. There have been many recent reviews on MMP,^149^^–^^151^ and so we do not describe it in detail here. In conclusion, polarized light imaging has proven to be a powerful tool for characterizing collagen architecture through different approaches and for understanding the biomechanics of soft tissue.

The imaging methods described here will continue to improve as the electronics and optics in the microscopes improve, such as increased frame rates and lower noise floors for cameras, or faster and more accurate stages. These advances will lead to faster image collection, particularly in the line- and point-scanning methods of PS-OCT and pSHG, creating greater opportunity for volumetric and *in vivo* imaging of dynamic processes, as shown in Fig. 18.^148^ These volumetric and *in vivo* capabilities could measure the acute mechanical response of the optic nerve head,^152^ arterial tissue,^153^ cervical tissue,^154^ vaginal tissue,^155^ or tendons and ligaments.^156^^,^^157^ These polarized light methods are also capable of tracking mechanical properties and remodeling of these tissues longitudinally with disease development and progression. Decreases in the costs of cameras and other electronics will also further stimulate the development of multicamera array microscopes,^158^ enabling PLM and IPOL imaging of larger fields of view at multiple length scales simultaneously, which would extend their capability of dynamic measurements. The above are elementary speculations of how the field will advance. However, as with other advances in science and technology, the most exciting developments will likely be those that we cannot predict simply from the extrapolation of current efforts. If the next few decades turn out to be even half as exciting as the past ones, it will be a fantastic ride.

## Data Availability

Data comparing AO PS-OCT and IPOL will be available upon request. All other results are a review of published material.

## References

[1] Shoulders M. D. Raines R. T., “Collagen structure and stability,” Annu. Rev. Biochem. 78(1), 929–958 (2009). ARBOAW 0066-4154 10.1146/annurev.biochem.77.032207.120833 19344236 PMC2846778

[r2] Sharabi M., “Structural mechanisms in soft fibrous tissues: a review,” Front. Mater. 8, 793647 (2022). 10.3389/fmats.2021.793647

[3] Siadat S. M. Ruberti J. W., “Mechanochemistry of collagen,” Acta Biomater. 163, 50–62 (2023). 10.1016/j.actbio.2023.01.025 36669548 PMC13270376

[4] Kalwani N. M. et al., “Quantitative polarized light microscopy of unstained mammalian cochlear sections,” J. Biomed. Opt. 18(2), 026021 (2013). JBOPFO 1083-3668 10.1117/1.JBO.18.2.026021 23407909 PMC3571355

[r5] Axer H. et al., “Quantitative estimation of 3-D fiber course in gross histological sections of the human brain using polarized light,” J. Neurosci. Methods 105(2), 121–131 (2001). JNMEDT 0165-0270 10.1016/S0165-0270(00)00349-6 11275269

[r6] Rieppo J. et al., “Practical considerations in the use of polarized light microscopy in the analysis of the collagen network in articular cartilage,” Microsc. Res. Tech. 71(4), 279–287 (2008). MRTEEO 1059-910X 10.1002/jemt.20551 18072283

[r7] Wolman M., “Polarized light microscopy as a tool of diagnostic pathology,” J. Histochem. Cytochem. 23(1), 21–50 (1975). 10.1177/23.1.1090645 1090645

[r8] Jan N.-J. et al., “Polarization microscopy for characterizing fiber orientation of ocular tissues,” Biomed. Opt. Express 6(12), 4705–4718 (2015). BOEICL 2156-7085 10.1364/BOE.6.004705 26713188 PMC4679248

[9] Menzel M. et al., “Diattenuation imaging reveals different brain tissue properties,” Sci. Rep. 9(1), 1939 (2019). 10.1038/s41598-019-38506-w 30760789 PMC6374401

[10] Mansfield J. C. et al., “Collagen fiber arrangement in normal and diseased cartilage studied by polarization sensitive nonlinear microscopy,” J. Biomed. Opt. 13(4), 044020 (2008). JBOPFO 1083-3668 10.1117/1.2950318 19021348

[r11] Brasselet S., “Polarization-resolved nonlinear microscopy: application to structural molecular and biological imaging,” Adv. Opt. Photonics 3(3), 205 (2011). AOPAC7 1943-8206 10.1364/AOP.3.000205

[r12] Golaraei A. et al., “Characterization of collagen in non-small cell lung carcinoma with second harmonic polarization microscopy,” Biomed. Opt. Express 5(10), 3562–3567 (2014). BOEICL 2156-7085 10.1364/BOE.5.003562 25360372 PMC4206324

[r13] McQuaid R. et al., “Second-harmonic reflection imaging of normal and accelerated corneal crosslinking using porcine corneas and the role of intraocular pressure,” Cornea 33(2), 125–130 (2014). CORNDB 0277-3740 10.1097/ICO.0000000000000015 24322797

[14] Gusachenko I. Latour G. Schanne-Klein M.-C., “Polarization-resolved second harmonic microscopy in anisotropic thick tissues,” Opt. Express 18(18), 19339–19352 (2010). OPEXFF 1094-4087 10.1364/OE.18.019339 20940829

[15] de Boer J. F. Hitzenberger C. K. Yasuno Y., “Polarization sensitive optical coherence tomography—a review [Invited],” Biomed. Opt. Express 8(3), 1838–1873 (2017). BOEICL 2156-7085 10.1364/BOE.8.001838 28663869 PMC5480584

[r16] Li Q. Sampson D. D. Villiger M., “In vivo imaging of the depth-resolved optic axis of birefringence in human skin,” Opt. Lett. 45(17), 4919–4922 (2020). OPLEDP 0146-9592 10.1364/OL.400855 32870890 PMC7535952

[r17] Liu X. et al., “Posterior scleral birefringence measured by triple-input polarization-sensitive imaging as a biomarker of myopia progression,” Nat. Biomed. Eng. 7(8), 986–1000 (2023). 10.1038/s41551-023-01062-w 37365268 PMC10427432

[r18] Strasswimmer J. et al., “Polarization-sensitive optical coherence tomography of invasive basal cell carcinoma,” J. Biomed. Opt. 9(2), 292–298 (2004). JBOPFO 1083-3668 10.1117/1.1644118 15065894

[r19] Tang P. et al., “Local axis orientation mapped by polarization sensitive optical coherence tomography provides a unique contrast to identify caries lesions in enamel,” Biomed. Opt. Express 13(8), 4247–4260 (2022). BOEICL 2156-7085 10.1364/BOE.464707 36032575 PMC9408248

[r20] de Boer J. F. et al., “Two-dimensional birefringence imaging in biological tissue by polarization-sensitive optical coherence tomography,” (in English), Opt. Lett. 22(12), 934–936 (1997). OPLEDP 0146-9592 10.1364/OL.22.000934 18185711

[r21] Schoenenberger K. et al. “Mapping of birefringence and thermal damage in tissue by use of polarization-sensitive optical coherence tomography,” Appl. Opt. 37(25), 6026–6036 (1998). APOPAI 0003-6935 10.1364/AO.37.006026 18286100

[22] Jiao S. Wang L. V., “Jones-matrix imaging of biological tissues with quadruple-channel optical coherence tomography,” (in English), J. Biomed. Opt. 7(3), 350–358 (2002). JBOPFO 1083-3668 10.1117/1.1483878 12175284

[23] Meek K. Fullwood N. J., “Corneal and scleral collagens—a microscopist’s perspective,” Micron 32(3), 261–272 (2001). MICNB2 0047-7206 10.1016/S0968-4328(00)00041-X 11006506

[24] Wang B. et al., “Collagen fiber interweaving is central to sclera stiffness,” Acta Biomater. 113, 429–437 (2020). 10.1016/j.actbio.2020.06.026 32585309 PMC7425206

[25] Lee P. Y. et al., “Real-time imaging of optic nerve head collagen microstructure and biomechanics using instant polarized light microscopy,” Exp. Eye Res. 217, 108967 (2022). EXERA6 0014-4835 10.1016/j.exer.2022.108967 35114213 PMC8957577

[26] Akhtar R. et al. “Characterizing the elastic properties of tissues,” Mater. Today 14(3), 96–105 (2011). MATOBY 1369-7021 10.1016/S1369-7021(11)70059-1 PMC3378034 22723736

[r27] Arkill K. P. Moger J. Winlove C. P., “The structure and mechanical properties of collecting lymphatic vessels: an investigation using multimodal nonlinear microscopy,” J. Anat. 216(5), 547–555 (2010). JOANAY 0021-8782 10.1111/j.1469-7580.2010.01215.x 20345855 PMC2871990

[r28] Chen H. et al., “Microstructure and mechanical property of glutaraldehyde-treated porcine pulmonary ligament,” J. Biomech. Eng. 138(6), 061003 (2016). JBENDY 0148-0731 10.1115/1.4033300 27040732 PMC7104758

[r29] Gao J. et al. “Relationship between microstructure, material distribution, and mechanical properties of sheep tibia during fracture healing process,” Int. J. Med. Sci. 10(11), 1560 (2013). 10.7150/ijms.6611 24046532 PMC3775115

[r30] Gong X. et al. “Alterations in biomechanical properties and microstructure of colon wall in early-stage experimental colitis,” Exp. Ther. Med. 14(2), 995–1000 (2017). 10.3892/etm.2017.4607 28810551 PMC5526050

[r31] Haut R. C., “Biomechanics of soft tissue,” in Accidental Injury, Nahum A. M. Melvin J. W., Eds., pp. 228–253, Springer (2002).

[r32] Holzapfel G. A. Ogden R. W., “Biomechanical relevance of the microstructure in artery walls with a focus on passive and active components,” Am. J. Physiol. Heart Circ. Physiol. 315(3), H540–H549 (2018). 10.1152/ajpheart.00117.2018 29799274

[r33] Kotova S. L. et al., “Collagen structure deterioration in the skin of patients with pelvic organ prolapse determined by atomic force microscopy,” Microsc. Microanal. 21(2), 324 (2015). MIMIF7 1431-9276 10.1017/S1431927615000148 25740571

[r34] Schwarcz H. P. Abueidda D. Jasiuk I., “The ultrastructure of bone and its relevance to mechanical properties,” Front. Phys. 5, 39 (2017). FRPHAY 0429-7725 10.3389/fphy.2017.00039

[r35] Voorhees A. Jan N.-J. Sigal I., “Effects of collagen microstructure and material properties on the deformation of the neural tissues of the lamina cribrosa,” Acta Biomater. 58, 278–290 (2017). 10.1016/j.actbio.2017.05.042 28528864 PMC5537032

[r36] Whitford C. et al., “Biomechanical model of the human cornea: considering shear stiffness and regional variation of collagen anisotropy and density,” J. Mech. Behav. Biomed. Mater. 42, 76–87 (2015). 10.1016/j.jmbbm.2014.11.006 25460928

[r37] Wright J. O. et al., “Microstructural and mechanical properties of the posterior cruciate ligament: a comparison of the anterolateral and posteromedial bundles,” J. Bone Joint Surg. Am. 98(19), 1656–1664 (2016). 10.2106/JBJS.16.00032 27707852

[38] Zarei V. et al., “Tissue loading and microstructure regulate the deformation of embedded nerve fibres: predictions from single-scale and multiscale simulations,” J. R. Soc. Interface 14(135), 20170326 (2017).1742-5689 10.1098/rsif.2017.0326 28978743 PMC5665822

[39] Elbischger P. et al., “Modeling and characterizing collagen fiber bundles,” in 3rd IEEE Int. Symp. Biomed. Imaging: Nano to Macro, IEEE, pp. 1280–1283 (2006). 10.1109/ISBI.2006.1625159

[40] Grytz R. Meschke G., “Microstructure-oriented modeling and computational remodeling of the Collagen Network in corneo-scleral shells,” in ECCOMAS Multidiscipl. Jubilee Symp., Springer, pp. 155–168 (2009).

[41] Yang B. et al., “Instant polarized light microscopy for imaging collagen microarchitecture and dynamics,” J. Biophotonics 14(2), e202000326 (2021). 10.1002/jbio.202000326 33103363 PMC7887070

[42] Roeder B. A. et al., “Tensile mechanical properties of three-dimensional type I collagen extracellular matrices with varied microstructure,” J. Biomech. Eng. 124(2), 214–222 (2002). JBENDY 0148-0731 10.1115/1.1449904 12002131

[43] Xu B. Chow M.-J. Zhang Y., “Experimental and modeling study of collagen scaffolds with the effects of crosslinking and fiber alignment,” Int. J. Biomater. 2011, 172389 (2011). 10.1155/2011/172389 21876695 PMC3162969

[44] Winkler M. et al., “Nonlinear optical macroscopic assessment of 3-D corneal collagen organization and axial biomechanics,” Invest. Ophthalmol. Vis. Sci. 52(12), 8818–8827 (2011). 10.1167/iovs.11-8070 22003117 PMC3230904

[45] Rajesh M. Singh S. P. Pitchaimani J., “Mechanical behavior of woven natural fiber fabric composites: effect of weaving architecture, intra-ply hybridization and stacking sequence of fabrics,” J. Ind. Text. 47(5), 938–959 (2018). 10.1177/1528083716679157

[46] Kastelic J. Palley I. Baer E., “A structural mechanical model for tendon crimping,” J. Biomech. 13(10), 887–893 (1980). JBMCB5 0021-9290 10.1016/0021-9290(80)90177-3 7462263

[47] Andreo R. Farrell R., “Corneal small-angle light-scattering theory: wavy fibril models,” JOSA 72(11), 1479–1492 (1982). JSDKD3 10.1364/JOSA.72.001479 7143127

[48] Brazile B. L. et al., “Thin lamina cribrosa beams have different collagen microstructure than thick beams,” Invest. Ophthalmol. Vis. Sci. 59(11), 4653–4661 (2018). IOVSDA 0146-0404 10.1167/iovs.18-24763 30372734 PMC6149225

[r49] Jan N. J. et al., “Crimp around the globe; patterns of collagen crimp across the corneoscleral shell,” Exp. Eye Res. 172, 159–170 (2018). EXERA6 0014-4835 10.1016/j.exer.2018.04.003 29660327 PMC5994193

[50] Jan N.-J. et al., “Microstructural crimp of the lamina cribrosa and peripapillary sclera collagen fibers,” Invest. Ophthalmol. Vis. Sci. 58(9), 3378–3388 (2017). 10.1167/iovs.17-21811 28687851 PMC5501496

[51] Collett E., Field Guide to Polarization, SPIE Press, Bellingham, Washington (2005).

[52] Wang L. Zimnyakov D., Optical Polarization in Biomedical Applications, Springer (2006).

[53] Izotova V. F. et al., “Investigation of Mueller matrices of anisotropic nonhomogeneous layers in application to an optical model of the cornea,” Appl. Opt. 36(1), 164–169 (1997). APOPAI 0003-6935 10.1364/AO.36.000164 18250658

[r54] Arokoski J. et al., “Decreased birefringence of the superficial zone collagen network in the canine knee (stifle) articular cartilage after long distance running training, detected by quantitative polarised light microscopy,” Ann. Rheum. Dis. 55(4), 253–264 (1996). ARDIAO 0003-4967 10.1136/ard.55.4.253 8733443 PMC1010147

[r55] Hemenger R. P., “Refractive index changes in the ocular lens result from increased light scatter,” J. Biomed. Opt. 1(3), 268–272 (1996). JBOPFO 1083-3668 10.1117/12.242695 23014725

[r56] Knighton R. W. Huang X.-R., “Linear birefringence of the central human cornea,” Invest. Ophthalmol. Vis. Sci. 43(1), 82–86 (2002). 11773016

[57] Alali S. Wang Y. Vitkin I. A., “Detecting axial heterogeneity of birefringence in layered turbid media using polarized light imaging,” Biomed. Opt. Express 3(12), 3250–3263 (2012). BOEICL 2156-7085 10.1364/BOE.3.003250 23243575 PMC3521317

[58] Maitland D. J. Walsh J. T. Jr., “Quantitative measurements of linear birefringence during heating of native collagen,” Lasers Surg. Med. 20(3), 310–318 (1997). LSMEDI 0196-8092 10.1002/(SICI)1096-9101(1997)20:3<310::AID-LSM10>3.0.CO;2-H 9138260

[r59] Simonenko G. Tuchin V. Lakodina N., “Measurement of the optical anisotropy of biological tissues with the use of a nematic liquid crystal cell,” J. Opt. Technol. 67(6), 559 (2000). JOTEE4 1070-9762 10.1364/JOT.67.000559

[60] Simonenko G. V. et al., “Measurement of an optical anisotropy of biotissues,” Proc. SPIE 3915, 152–157 (2000). PSISDG 0277-786X 10.1117/12.384167

[61] Sadat-Shojai M. et al., “Synthesis methods for nanosized hydroxyapatite with diverse structures,” Acta Biomater. 9(8), 7591–7621 (2013). 10.1016/j.actbio.2013.04.012 23583646

[62] Emoto A. et al., “Form birefringence in intrinsic birefringent media possessing a subwavelength structure,” Appl. Opt. 49(23), 4355–4361 (2010). APOPAI 0003-6935 10.1364/AO.49.004355 20697436

[63] Menzel M. et al., “A Jones matrix formalism for simulating three-dimensional polarized light imaging of brain tissue,” J. R. Soc. Interface 12(111), 20150734 (2015).1742-5689 10.1098/rsif.2015.0734 26446561 PMC4614509

[64] Wolman M. Kasten F., “Polarized light microscopy in the study of the molecular structure of collagen and reticulin,” Histochemistry 85(1), 41–49 (1986). HCMYAL 0301-5564 10.1007/BF00508652 3733471

[65] Hemenger R. P., “Birefringence of a medium of tenuous parallel cylinders,” Appl. Opt. 28(18), 4030–4034 (1989). APOPAI 0003-6935 10.1364/AO.28.004030 20555816

[66] De Boer J. F. Milner T. E., “Review of polarization sensitive optical coherence tomography and Stokes vector determination,” J. Biomed. Opt. 7(3), 359–371 (2002). JBOPFO 1083-3668 10.1117/1.1483879 12175285

[67] Born M. Wolf E., Principles of Optics: Electromagnetic Theory of Propagation, Interference and Diffraction of Light, Elsevier (2013).

[68] Spiesz E. M. Kaminsky W. Zysset P. K., “A quantitative collagen fibers orientation assessment using birefringence measurements: calibration and application to human osteons,” J. Struct. Biol. 176(3), 302–306 (2011). JSBIEM 1047-8477 10.1016/j.jsb.2011.09.009 21970947 PMC3218218

[69] Yang B. et al., “Polarized light microscopy for 3‐dimensional mapping of collagen fiber architecture in ocular tissues,” J. Biophotonics 11(8), e201700356 (2018). 10.1002/jbio.201700356 29633576 PMC6105457

[70] Schmidt W. J., Die Bausteine des Tierkörpers in polarisiertem Lichte, F. Cohen (1924).

[71] Schmidt W. J., Die doppelbrechung von karyoplasma, zytoplasma und metaplasma, Gebrüder Bornträger (1937).

[72] Gustafson A.-G., “A new method of microphotography of dental enamel in polarised light,” Acta Odontol. Scand. 25(6), 647–655 (1967). AOSCAQ 0001-6357 10.3109/00016356709019781 5247244

[73] Diamant J. et al., “Collagen; ultrastructure and its relation to mechanical properties as a function of ageing,” Proc. R. Soc. Lond. Ser. B. Biol. Sci. 180(1060), 293–315 (1972). 10.1098/rspb.1972.0019 4402469

[74] Glazer A. Lewis J. Kaminsky W., “An automatic optical imaging system for birefringent media,” Proc. R. Soc. Lond. Ser. A: Math. Phys. Eng. Sci. 452(1955), 2751–2765 (1996). 10.1098/rspa.1996.0145

[75] Baba J. S. Cameron B. D. Cote G. L., “Effect of temperature, pH, and corneal birefringence on polarimetric glucose monitoring in the eye,” J. Biomed. Opt. 7(3), 321–328 (2002). JBOPFO 1083-3668 10.1117/1.1484163 12175281

[76] Jan N.-J. Lathrop K. Sigal I. A., “Collagen architecture of the posterior pole: high-resolution wide field of view visualization and analysis using polarized light microscopy,” Invest. Ophthalmol. Vis. Sci. 58(2), 735–744 (2017). 10.1167/iovs.16-20772 28146238 PMC5295768

[77] Shribak M. Oldenbourg R., “Techniques for fast and sensitive measurements of two-dimensional birefringence distributions,” Appl. Opt. 42(16), 3009–3017 (2003). APOPAI 0003-6935 10.1364/AO.42.003009 12790452

[78] Raub C. et al., “Microstructural remodeling of articular cartilage following defect repair by osteochondral autograft transfer,” Osteoarthr. Cartil. 21(6), 860–868 (2013). OSCAEO 1063-4584 10.1016/j.joca.2013.03.014 PMC3684698 23528954

[79] Sekita A. Matsugaki A. Nakano T., “Disruption of collagen/apatite alignment impairs bone mechanical function in osteoblastic metastasis induced by prostate cancer,” Bone 97, 83–93 (2017). 10.1016/j.bone.2017.01.004 28069516

[80] Madibally S. V. et al., “Influence of insulin therapy on burn wound healing in rats,” J. Surg. Res. 109(2), 92–100 (2003). 10.1016/S0022-4804(02)00036-7 12643849

[81] Rowe A. J. Finlay H. M. Canham P. B., “Collagen biomechanics in cerebral arteries and bifurcations assessed by polarizing microscopy,” J. Vasc. Res. 40(4), 406–415 (2003). JVREE9 1018-1172 10.1159/000072831 12913333

[82] Axer M. et al., “High-resolution fiber tract reconstruction in the human brain by means of three-dimensional polarized light imaging,” (in English), Front. Neuroinf. 5, 34 (2011). 10.3389/fninf.2011.00034 PMC3248698 22232597

[83] Allen R. D. Brault J. Moore R. D., “A new method of polarization microscopic analysis: I. scanning with a birefringence detection system,” J. Cell Biol. 18(2), 223–235 (1963). JCLBA3 0021-9525 10.1083/jcb.18.2.223 14079486 PMC2106293

[84] Kuhn J. R. Wu Z. Poenie M., “Modulated polarization microscopy: a promising new approach to visualizing cytoskeletal dynamics in living cells,” Biophys. J. 80(2), 972–985 (2001). BIOJAU 0006-3495 10.1016/S0006-3495(01)76076-6 11159464 PMC1301295

[85] Keikhosravi A. et al., “Quantification of collagen organization in histopathology samples using liquid crystal based polarization microscopy,” Biomed. Opt. Express 8(9), 4243–4256 (2017). BOEICL 2156-7085 10.1364/BOE.8.004243 28966862 PMC5611938

[r86] Mehta S. B. Shribak M. Oldenbourg R., “Polarized light imaging of birefringence and diattenuation at high resolution and high sensitivity,” J. Opt. 15(9), 094007 (2013). JOOPDB 0150-536X 10.1088/2040-8978/15/9/094007 PMC3834771 24273640

[87] Oldenbourg R. Mei G., “New polarized light microscope with precision universal compensator,” J. Microsc. 180(2), 140–147 (1995). JMICAR 0022-2720 10.1111/j.1365-2818.1995.tb03669.x 8537959

[88] Shribak M. et al., “Instantaneous polarization measurement system and method,” U.S. Patent No. 7,079,247 (2006).

[89] Kaminsky W. et al., “Simultaneous false‐colour imaging of birefringence, extinction and transmittance at camera speed,” J. Microsc. 228(2), 153–164 (2007). JMICAR 0022-2720 10.1111/j.1365-2818.2007.01841.x 17970915

[90] Gruev V. Perkins R. York T., “CCD polarization imaging sensor with aluminum nanowire optical filters,” Opt. Express 18(18), 19087–19094 (2010). OPEXFF 1094-4087 10.1364/OE.18.019087 20940803

[91] York T. et al., “Bioinspired polarization imaging sensors: from circuits and optics to signal processing algorithms and biomedical applications,” Proc. IEEE 102(10), 1450–1469 (2014). IEEPAD 0018-9219 10.1109/JPROC.2014.2342537 PMC4629637 26538682

[92] York T. et al., “Real-time high-resolution measurement of collagen alignment in dynamically loaded soft tissue,” J. Biomed. Opt. 19(6), 066011 (2014). JBOPFO 1083-3668 10.1117/1.JBO.19.6.066011 24972359

[93] Shribak M., “Polychromatic polarization microscope: bringing colors to a colorless world,” Sci. Rep. 5, 17340 (2015). 10.1038/srep17340 26611150 PMC4661494

[94] Yamanari M. et al., “Scleral birefringence as measured by polarization-sensitive optical coherence tomography and ocular biometric parameters of human eyes in vivo,” Biomed. Opt. Express 5(5), 1391–1402 (2014). BOEICL 2156-7085 10.1364/BOE.5.001391 24877003 PMC4026890

[95] Lee P. Y. et al., “Instant polarized light microscopy pi (IPOLpi) for quantitative imaging of collagen architecture and dynamics in ocular tissues,” Opt. Lasers Eng. 166, 107594 (2023). 10.1016/j.optlaseng.2023.107594 37193214 PMC10168649

[96] Yang B. et al., “Structured polarized light microscopy for collagen fiber structure and orientation quantification in thick ocular tissues,” J. Biomed. Opt. 23(10), 106001 (2018). JBOPFO 1083-3668 10.1117/1.JBO.23.10.106001 30277032 PMC6210789

[97] Neil M. A. Juškaitis R. Wilson T., “Method of obtaining optical sectioning by using structured light in a conventional microscope,” Opt. Lett. 22(24), 1905–1907 (1997). OPLEDP 0146-9592 10.1364/OL.22.001905 18188403

[98] Saxena M. Eluru G. Gorthi S. S., “Structured illumination microscopy,” Adv. Opt. Photonics 7(2), 241–275 (2015). AOPAC7 1943-8206 10.1364/AOP.7.000241

[99] Huang D. et al., “Optical coherence tomography,” (in English), Science 254(5035), 1178–1181 (1991). SCIEAS 0036-8075 10.1126/science.1957169 1957169 PMC4638169

[100] Aumann S. et al., “Optical coherence tomography (OCT): principle and technical realization,” in High Resolution Imaging in Microscopy and Ophthalmology: New Frontiers in Biomedical Optics, Bille J. F. Ed., pp. 59–85, Cham (2019).

[r101] Bouma B. E. et al., “Optical coherence tomography,” Nat. Rev. Methods Primers 2, 80 (2022). 10.1038/s43586-022-00162-2 PMC9901537 36751306

[102] Drexler W. Fujimoto J. G., Eds. Optical Coherence Tomography: Technology and Applications, 2nd ed., SpringerReference, Berlin (2015).

[103] Wang Y. et al., “Application of optical coherence tomography in clinical diagnosis,” J. X-Ray Sci. Technol. 27(6), 995–1006 (2019). JXSTE5 0895-3996 10.3233/XST-190559 PMC7029333 31594279

[104] Masoumi S. et al., “Absolute depth-resolved optic axis measurement with catheter-based polarization sensitive optical coherence tomography,” Biomed. Opt. Express 15(12), 6957–6976 (2024). BOEICL 2156-7085 10.1364/BOE.538560 39679397 PMC11640563

[105] Nandy S. et al., “Polarization-sensitive endobronchial optical coherence tomography for microscopic imaging of fibrosis in interstitial lung disease,” Am. J. Respir. Crit. Care Med. 206(7), 905–910 (2022). AJCMED 1073-449X 10.1164/rccm.202112-2832LE 35675552 PMC12042782

[106] Everett M. J. et al., “Birefringence characterization of biological tissue by use of optical coherence tomography,” Opt. Lett. 23(3), 228–230 (1998). OPLEDP 0146-9592 10.1364/OL.23.000228 18084468

[r107] Hee M. R. et al., “Polarization-sensitive low-coherence reflectometer for birefringence characterization and ranging,” J. Opt. Soc. Am. B 9(6), 903–908 (1992). JOBPDE 0740-3224 10.1364/JOSAB.9.000903

[108] Hitzenberger C. K. et al., “Measurement and imaging of birefringence and optic axis orientation by phase resolved polarization sensitive optical coherence tomography,” Opt. Express 9(13), 780–790 (2001). OPEXFF 1094-4087 10.1364/OE.9.000780 19424315

[109] Yao G. Wang L. V., “Two-dimensional depth-resolved Mueller matrix characterization of biological tissue by optical coherence tomography,” Opt. Lett. 24(8), 537–539 (1999). OPLEDP 0146-9592 10.1364/OL.24.000537 18071564

[110] Baumann B., “Polarization sensitive optical coherence tomography: a review of technology and applications,” Appl. Sci. 7(5), 474 (2017). 10.3390/app7050474

[111] Dwelle J. et al., “Thickness, phase retardation, birefringence, and reflectance of the retinal nerve fiber layer in normal and glaucomatous non-human primates,” Invest. Ophthalmol. Vis. Sci. 53(8), 4380–4395 (2012). IOVSDA 0146-0404 10.1167/iovs.11-9130 22570345 PMC3394663

[r112] Afsharan H. et al., “Hypertension-associated changes in retinal blood vessel walls measured in vivo with polarization-sensitive optical coherence tomography,” Opt. Lasers Eng. 172 (2024). OLENDN 0143-8166 10.1016/j.optlaseng.2023.107838

[r113] Hong Y.-J. et al., “Simultaneous investigation of vascular and retinal pigment epithelial pathologies of exudative macular diseases by multifunctional optical coherence tomography,” Invest. Ophthalmol. Vis. Sci. 55(8), 5016–5031 (2014). 10.1167/iovs.14-14005 25052993

[r114] Parakkel R. R. et al., “Retinal nerve fiber layer damage assessment in glaucomatous eyes using retinal retardance measured by polarization-sensitive optical coherence tomography,” Transl. Vis. Sci. Technol. 13(5), 9 (2024). 10.1167/tvst.13.5.9 PMC11103739 38743409

[r115] Pollreisz A. et al., “Early identification of retinal neuropathy in subclinical diabetic eyes by reduced birefringence of the peripapillary retinal nerve fiber layer,” Invest. Ophthalmol. Vis. Sci. 62(4), 24–24 (2021). 10.1167/iovs.62.4.24 PMC8083066 33871570

[116] Steiner S. et al., “Birefringent properties of the peripapillary retinal nerve fiber layer in healthy and glaucoma subjects analyzed by polarization-sensitive OCT,” Invest. Ophthalmol. Vis. Sci. 63(12), 8 (2022). 10.1167/iovs.63.12.8 PMC9645361 36331260

[117] Sakata L. M. et al., “Optical coherence tomography of the retina and optic nerve—a review,” Clin. Exp. Ophthalmol. 37(1), 90–99 (2009). 10.1111/j.1442-9071.2009.02015.x 19338607

[118] Kurokawa K. Nemeth M., “Multifunctional adaptive optics optical coherence tomography allows cellular scale reflectometry, polarimetry, and angiography in the living human eye,” Biomed. Opt. Express 15(2), 1331–1354 (2024). BOEICL 2156-7085 10.1364/BOE.505395 38404344 PMC10890865

[119] Xiong Q. et al., “Constrained polarization evolution simplifies depth-resolved retardation measurements with polarization-sensitive optical coherence tomography,” Biomed. Opt. Express 10(10), 5207–5222 (2019). BOEICL 2156-7085 10.1364/BOE.10.005207 31646042 PMC6788597

[120] Tang P. Wang R. K., “Polarization state tracing method to map local birefringent properties in samples using polarization sensitive optical coherence tomography,” Biomed. Opt. Express 11(12), 6852–6863 (2020). BOEICL 2156-7085 10.1364/BOE.408667 33408966 PMC7747894

[121] Makita S. Yamanari M. Yasuno Y., “Generalized Jones matrix optical coherence tomography: performance and local birefringence imaging,” Opt. Express 18(2), 854–876 (2010). OPEXFF 1094-4087 10.1364/OE.18.000854 20173907

[122] Li Q. et al., “Robust reconstruction of local optic axis orientation with fiber-based polarization-sensitive optical coherence tomography,” Biomed. Opt. Express 9(11), 5437–5455 (2018). BOEICL 2156-7085 10.1364/BOE.9.005437 30460138 PMC6238922

[123] Chen X. Y. et al., “Second harmonic generation microscopy for quantitative analysis of collagen fibrillar structure,” (in English), Nat. Protoc. 7(4), 654–669 (2012).1754-2189 10.1038/nprot.2012.009 22402635 PMC4337962

[124] Bancelin S. et al., “Determination of collagen fibril size via absolute measurements of second-harmonic generation signals,” Nat. Commun. 5, 4920 (2014). NCAOBW 2041-1723 10.1038/ncomms5920 25223385

[125] Raoux C. et al., “Unveiling the lamellar structure of the human cornea over its full thickness using polarization-resolved SHG microscopy,” Light: Sci. Appl. 12(1), 190 (2023). 10.1038/s41377-023-01224-0 37528091 PMC10394036

[r126] Stoller P. et al., “Polarization-modulated second harmonic generation in collagen,” Biophys. J. 82(6), 3330–3342 (2002). 10.1016/S0006-3495(02)75673-7 12023255 PMC1302120

[r127] Tiaho F. Recher G. Rouède D., “Estimation of helical angle of myosin and collagen by second harmonic generation imaging microscopy ” Opt. Express 15(19), 12286–12295 (2007). OPEXFF 1094-4087 10.1364/OE.15.012286 19547597

[r128] Tuer A. E. et al., “Hierarchical model of fibrillar collagen organization for interpreting the second-order susceptibility tensors in biological tissue,” (in English), Biophys. J. 103(10), 2093–2105 (2012). BIOJAU 0006-3495 10.1016/j.bpj.2012.10.019 23200043 PMC3512050

[r129] Duboisset J. et al., “Generic model of the molecular orientational distribution probed by polarization-resolved second-harmonic generation,” (in English), Phys. Rev. A 85(4), (2012). 10.1103/PhysRevA.85.043829

[r130] Avila F. J. Del Barco O. Bueno J. M., “Polarization dependence of aligned collagen tissues imaged with second harmonic generation microscopy,” J. Biomed. Opt. 20(8), 086001 (2015). JBOPFO 1083-3668 10.1117/1.JBO.20.8.086001 26263415

[r131] Romijn E. I. Finnoy A. Lilledahl M. B., “Analyzing the feasibility of discriminating between collagen types I and II using polarization-resolved second harmonic generation,” (in English), J. Biophotonics 12(1), e201800090 (2019). 10.1002/jbio.201800090 29971934

[132] Mercatelli R. et al., “Collagen ultrastructural symmetry and its malignant alterations in human breast cancer revealed by polarization-resolved second-harmonic generation microscopy,” J. Biophotonics 13(8), e202000159 (2020). 10.1002/jbio.202000159 32472568

[133] Samim M. Krouglov S. Barzda V., “Double Stokes Mueller polarimetry of second-harmonic generation in ordered molecular structures,” (in English), J. Opt. Soc. Am. B: Opt. Phys. 32(3), 451–461 (2015). 10.1364/JOSAB.32.000451

[134] Teulon C. et al., “Theoretical, numerical and experimental study of geometrical parameters that affect anisotropy measurements in polarization-resolved SHG microscopy,” Opt. Express 23(7), 9313–9328 (2015). OPEXFF 1094-4087 10.1364/OE.23.009313 25968762

[135] Tuer A. E. et al., “Nonlinear optical properties of Type I collagen fibers studied by polarization dependent second harmonic generation microscopy,” (in English), J. Phys. Chem. B 115(44), 12759–12769 (2011). JPCBFK 1520-6106 10.1021/jp206308k 21970315

[r136] Gusachenko I. et al., “Polarization-resolved second-harmonic microscopy in tendon upon mechanical stretching,” Biophys. J. 102(9), 2220–2229 (2012). BIOJAU 0006-3495 10.1016/j.bpj.2012.03.068 22824287 PMC3341536

[137] Galante G. et al., “Intrinsic variation of the polarization-resolved SHG from collagen: multiscale analysis and application to parchments,” APL Photonics 10(5) (2025). 10.1063/5.0250484

[138] Psilodimitrakopoulos S. et al, “Quantitative discrimination between endogenous SHG sources in mammalian tissue, based on their polarization response,” (in English), Opt. Express 17(12), 10168–10176 (2009). OPEXFF 1094-4087 10.1364/OE.17.010168 19506670

[139] Asadipour B. et al., “Modelling and predicting second harmonic generation from protein molecular structure,” Phys. Rev. X 14(1), 011038 (2024). PRXHAE 2160-3308 10.1103/PhysRevX.14.011038

[140] Tanaka Y. et al., “Motion-artifact-robust, polarization-resolved second-harmonic-generation microscopy based on rapid polarization switching with electro-optic Pockells cell and its application to in vivo visualization of collagen fiber orientation in human facial skin,” (in English), Biomed. Opt. Express 5(4), 1099–1113 (2014). BOEICL 2156-7085 10.1364/BOE.5.001099 24761292 PMC3985985

[r141] DeWalt E. L. et al., “Polarization-modulated second harmonic generation ellipsometric microscopy at video rate,” (in English), Anal. Chem. 86(16), 8448–8456 (2014). ANCHAM 0003-2700 10.1021/ac502124v 25050448 PMC4139166

[142] Ducourthial G. et al., “Monitoring dynamic collagen reorganization during skin stretching with fast polarization-resolved SHG imaging,” J. Biophotonics 12(5), e201800336 (2019). 10.1002/jbio.201800336 30604478

[143] Chen X. Raggio C. Campagnola P. J., “Second harmonic generation circular dichroism studies of osteogenesis imperfecta,” Opt. Lett. 37(18), 3837–3839 (2012). OPLEDP 0146-9592 10.1364/OL.37.003837 23041876 PMC4337953

[r144] Lee H. et al., “Chiral imaging of collagen by second-harmonic generation circular dichroism,” Biomed. Opt. Express 4(6), 909–916 (2013). BOEICL 2156-7085 10.1364/BOE.4.000909 23761852 PMC3675869

[r145] Golaraei A. et al., “Collagen chirality and three-dimensional orientation studied with polarimetric second-harmonic generation microscopy,” (in English), J. Biophotonics 12(1) (2019). 10.1002/jbio.201800241 30288949

[146] Schmeltz M. et al., “Circular dichroism-SHG microscopy probes the polarity distribution of collagen fibrils,” Optica 7(11), 1469–1476 (2020). 10.1364/OPTICA.399246

[147] Harvey M. et al., “High numerical aperture imaging allows chirality measurement in individual collagen fibrils using polarization second harmonic generation microscopy,” Nanophotonics 12(11), 2061–2071 (2023). 10.1515/nanoph-2023-0177 37215945 PMC10193268

[148] Benoit A. et al., “Simultaneous microstructural and mechanical characterization of human corneas at increasing pressure,” J. Mech. Behav. Biomed. Mater. 60, 93–105 (2016). 10.1016/j.jmbbm.2015.12.031 26773650

[149] Ahmad I. et al., “Mueller matrix polarimetry for characterization of skin tissue samples: a review,” Photodiagn. Photodyn. Ther. 31, 101708 (2020). 10.1016/j.pdpdt.2020.101708 32145374

[r150] He H. et al., “Mueller matrix polarimetry—an emerging new tool for characterizing the microstructural feature of complex biological specimen,” J. Lightwave Technol. 37(11), 2534–2548 (2018). JLTEDG 0733-8724 10.1109/JLT.2018.2868845

[151] Ghosh N. Vitkin I. A., “Tissue polarimetry: concepts, challenges, applications, and outlook,” J. Biomed. Opt. 16(11), 110801 (2011). JBOPFO 1083-3668 10.1117/1.3652896 22112102

[152] Wei J. et al., “Comparing acute IOP-induced lamina cribrosa deformations premortem and postmortem,” Transl. Vis. Sci. Technol. 11(12), 1 (2022). 10.1167/tvst.11.12.1 PMC9728494 36454578

[153] Agrawal Y. et al., “Effect of collagen fiber tortuosity distribution on the mechanical response of arterial tissues,” J. Biomech. Eng. 147(2), 021004 (2025). JBENDY 0148-0731 10.1115/1.4067152 39545747 PMC11748964

[154] Barnum C. E. et al., “Tensile mechanical properties and dynamic collagen fiber re-alignment of the murine cervix are dramatically altered throughout pregnancy,” J. Biomech. Eng. 139(6), 0610081 (2017). JBENDY 0148-0731 10.1115/1.4036473 28418563 PMC6993786

[155] Chi N. et al., “Distinctive structure, composition and biomechanics of collagen fibrils in vaginal wall connective tissues associated with pelvic organ prolapse,” Acta Biomater. 152, 335–344 (2022). 10.1016/j.actbio.2022.08.059 36055614 PMC10182770

[156] Smith M. V. et al., “Mechanical properties and microstructural collagen alignment of the ulnar collateral ligament during dynamic loading,” Am. J. Sports Med. 47(1), 151–157 (2019). 10.1177/0363546518812416 30495972

[157] Wu X. et al., “High-speed polarization imaging of dynamic collagen fiber realignment in tendon-to-bone insertion region,” J. Biomed. Opt. 23(11), 116002 (2018). JBOPFO 1083-3668 10.1117/1.JBO.23.11.116002 30392198

[158] Thomson E. E. et al., “Gigapixel imaging with a novel multi-camera array microscope,” eLife 11, e74988 (2022). 10.7554/eLife.74988 36515989 PMC9917455

